# Toxicity and toxicokinetics of the ethanol extract of Zuojin formula

**DOI:** 10.1186/s12906-022-03684-0

**Published:** 2022-08-15

**Authors:** Shuo Wang, Tao Zhang, Xiaoyan Liu, Zheng Yang, Ludi Li, Danping Shan, Yadong Gao, Yingzi Li, Yanying Li, Youbo Zhang, Qi Wang

**Affiliations:** 1 Department of Toxicology, School of Public Health, Peking University, No. 38 Xueyuan Road, Haidian District, Beijing, 100191 People’s Republic of China; 2 TCM R&D Center, Beijing Increase Pharm Co. Ltd., Beijing, 102200 China; 3 State Key Laboratory of Natural and Biomimetic Drugs, Department of Natural Medicines, School of Pharmaceutical Sciences, Peking University, Beijing, 100191 China; 4 Key Laboratory of State Administration of Traditional Chinese Medicine (TCM) for Compatibility Toxicology, Beijing, 100191 China; 5 Beijing Key Laboratory of Toxicological Research and Risk Assessment for Food Safety, Beijing, 100191 China

**Keywords:** Zuojin formula, Toxicity, Berberine, Spleen, Gastrointestinal

## Abstract

**Background:**

Zuojin formula, a traditional Chinese medicine, comprises *Coptis chinensis* and *Evodia rutaecarpa*. In our previous study, the total alkaloid extract from Zuojin formula (TAZF) showed potent and improved efficacy. However, its safety and toxicokinetics remain unknown. The objective of this study was to evaluate the safety of repeated administrations of TAZF and investigate the internal exposure of the main components and its relationship with toxic symptoms.

**Methods:**

Sprague–Dawley rats were orally administered TAZF at 0.4, 1.2 and 3.7 g/kg for 28 days, which was followed by a 14-day recovery period. The toxic effects were evaluated weekly by assessing body weight changes, food intake, blood biochemistry and haematological indices, organ weights and histological changes. A total of eight components were detected, including berberine, coptisine, epiberberine, palmatine, jatrorrhizine, columbamine, evodiamine, and rutaecarpine. The toxicokinetic profiles of the eight components were investigated after single and repeated administrations. Linear mixed effect models were applied to analyse the associations between internal exposure and toxic symptoms. Network pharmacology analysis was applied to explore the potential toxic mechanisms.

**Results:**

Compared with the vehicle group, the rats in the low- and medium-dose groups did not show noticeable abnormal changes, while rats in the high-dose group exhibited inhibition of weight gain, a slight reduction in food consumption, abdominal bloating and atrophy of the splenic white pulp during drug administration. The concentration of berberine in plasma was the highest among all compounds. Epiberberine was found to be associated with the inhibition of weight gain. Network pharmacology analysis suggested that the alkaloids might cause abdominal bloating by affecting the proliferation of smooth muscle cells. The benchmark dose lower confidence limits (based on body weight inhibition) of TAZF were 1.27 g/kg (male) and 1.91 g/kg (female).

**Conclusions:**

TAZF has no notable liver or kidney toxicity but carries risks of gastrointestinal and immune toxicity at high doses. Alkaloids from *Coptis chinensis* are the main plasma components related to the toxic effects of TAZF.

**Supplementary Information:**

The online version contains supplementary material available at 10.1186/s12906-022-03684-0.

## Background

Zuojin formula is a traditional Chinese medicine (TCM) comprised of *Coptis chinensis* (the dry roots of *Coptis chinensis* Franch.) and *Evodia rutaecarpa* (the dry mature fruits of *Euodia rutaecarpa* (Juss.) Benth.) at a ratio of 6:1. Zuojin formula is famous for its therapeutic effect against some digestive tract diseases, such as gastric acid reflux, stomachache, diarrhoea, and gastritis [1–4]. Zuojin formula can also exhibit antitumour and antidepressive effects [1, 5–9]. As a traditional formula with a long history, Zuojin Pill has been regarded as a safe and nontoxic drug, and there are few reports of adverse reactions.

However, *Evodia rutaecarpa*, one of the ingredients in Zuojin formula, has been found to have noticeable hepatotoxicity. Both water and alcohol extracts of *Evodia rutaecarpa* can lead to liver injury in a time- and dose-dependent manner [10–13]. Alkaloids, such as evodiamine and rutaecarpine, and limonoids in *Evodia rutaecarpa* are the main active components [14]. Since Zuojin Pill consists of these potential hepatotoxic components, it has a potential risk of toxicity.

In our previous study, the alkaloids in *Coptis chinensis* and Evodia *rutaecarpa* were enriched after being extracted and eluted with different concentrations of ethanol. The total alkaloid extract of Zuojin formula (TAZF) had better efficacy than Zuojin Pill, as TAZF administered at a dosage of 1.37 g/day (humans) or 0.12 g/kg (rats) exerted the same or better treatment effects against gastritis as 6 ~ 12 g/day Zuojin Pill (unpublished data). Our preliminary study found that the LD_50_ of TAZF in rats was over 5 g/kg, rating this formula as nontoxic (unpublished data). However, the toxicity of repeated TAZF treatment remains unknown. In the present study, we evaluated the safety and toxicokinetic profiles of TAZF in a 28-day oral toxicity study in rats. Furthermore, we intended to find the links between exposure and toxic symptoms using statistical modelling.

## Methods

### Materials, reagents and animals

Carbamazepine (CAS No.: 298–46-4), berberine (CAS No.: 2086-83-1), coptisine (CAS No.: 6020-18-4), epiberberine (CAS No.: 6873-09-2), palmatine (CAS No.: 3486-67-7), jatrorrhizine (CAS No.: 3621-38-3), columbamine (CAS No.: 483–34-1), evodiamine (CAS No.: 518–17-2) and rutaecarpine (CAS No.: 20575–76-2), all > 98% purity, were purchased from Shanghai Yuanye Bio-Technology Company. Acetonitrile and methanol (LC grade) were purchased from Fisher Chemical, USA. Formaldehyde (AR) was purchased from Beijing Tong Guang Fine Chemicals Company. Isoflurane was provided by HARVEYBIO Company.

TAZF was provided by the State Key Laboratory of Natural and Biomimetic Drugs, School of Pharmaceutical Sciences, Peking University. The concentrations of the compounds in TAZF were measured using high-performance liquid chromatography (HPLC), with berberine accounting for the majority of TAZF at a ratio of 14.73% (g/g), followed by palmatine (3.14%), coptisine (2.52%), evodiamine (1.41%), epiberberine (1.30%), rutaecarpine (1.27%), columbamine (0.90%) and jatrorrhizine (0.47%).

### Repeated administration toxicity test

#### Animal administration and treatments

Sprague–Dawley (SD) rats (5 ~ 6 weeks old, male: 146.5 ± 7.9 g, female: 141.1 ± 9.1 g) were obtained from the Department of Laboratory Animal Science, Peking University Health Science Center. Animals were acclimated in environmentally controlled rooms (temperature of 20 ~ 25 °C; relative humidity of 50 ~ 55%; 12-h light/dark cycle) with food and water available ad libitum for approximately 1 week prior to the initiation of dosing. All experimental procedures were carried out following the National Institutes of Health Guide for the Care and Use of Laboratory Animals. Animal experimental protocols were approved by the Peking University Medical Ethical Review Committee (No. LA2018313).

Rats were randomly allocated to control and treated groups (16/group/sex), and administered the appropriate material via gavage for 28 days, which could support a 2-week clinical therapy regimen according to ICH guidelines [15–17]. Rats in the vehicle control group were treated with deionized water, while those in the treated groups were administered TAZF at dosages of 3.7 g/kg (high dose), 1.2 g/kg (medium dose), and 0.4 g/kg (low dose), which were close to 30-, 10-, and 3-fold the therapeutic dose in rats. The possible dosages of the main components are listed in Table 1.

**Table 1:** The dosage of main components in TAZF administered by gavage in rats

Dose	TAZF (g/kg)	Berberine (mg/kg)	Epiberbrine (mg/kg)	Coptisine (mg/kg)	Palmatine (mg/kg)	Jatrorrhizine (mg/kg)	Columbamine (mg/kg)	Evodiamine (mg/kg)	Rutecarpine (mg/kg)
High	3.7	545.01	50.69	93.24	116.18	17.39	35.15	52.17	46.99
Medium	1.2	176.76	16.44	30.24	37.68	5.64	11.40	16.92	15.24
Low	0.4	58.92	5.48	10.08	12.56	1.88	3.80	5.64	5.08

**Fig. 1:**
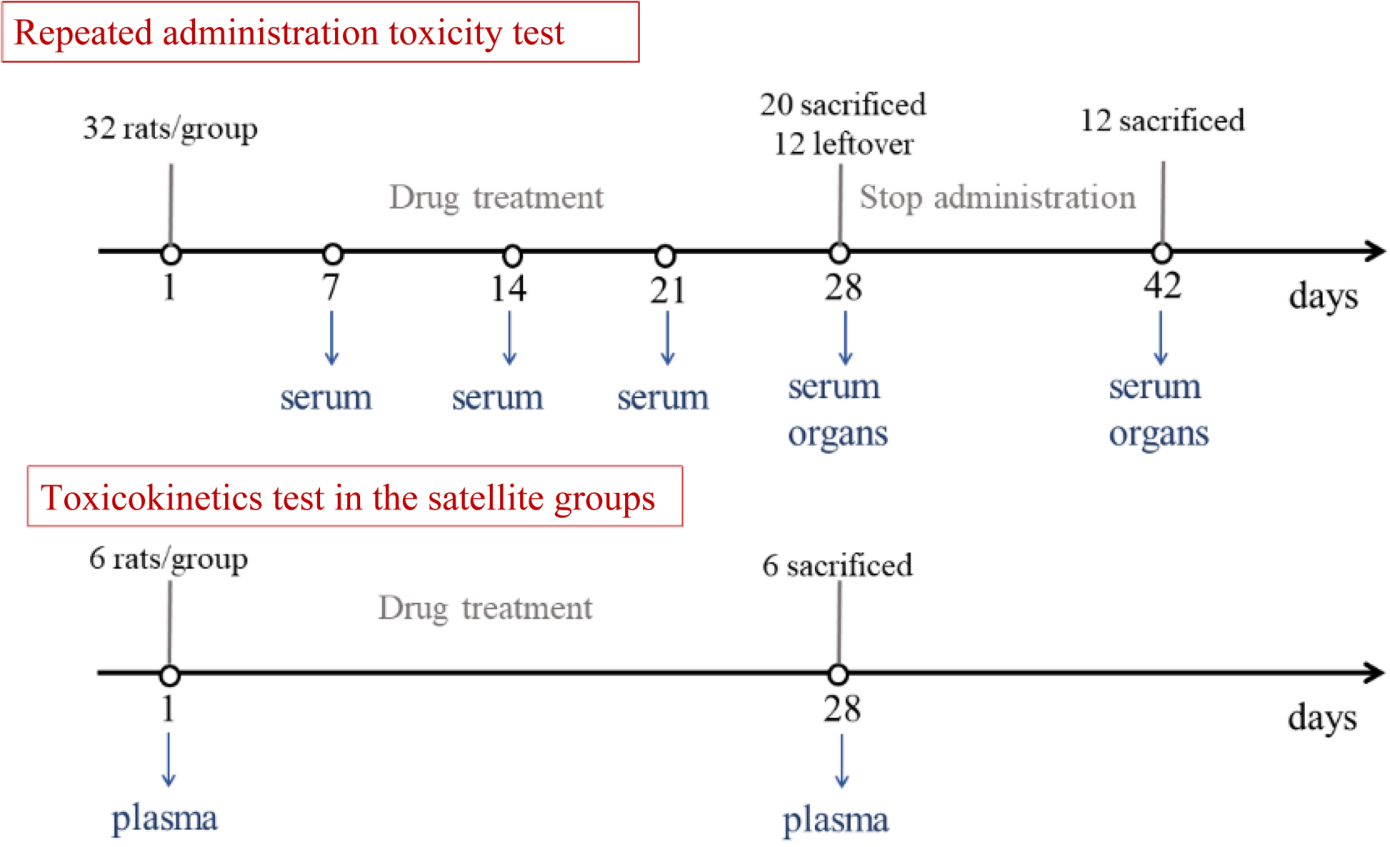
Animal treatment schedule

As shown in Fig. 1, blood was collected on the 7th, 14th^,^ and 21st days for biochemical analysis. On the 29th day, twenty rats from each group were sacrificed under isoflurane anaesthesia. Blood was collected, and the main organs were weighed and fixed in formalin. Organ coefficients (OCs) ($\mathrm{OCs}=\frac{\mathrm{organ}\ \mathrm{weight}}{\mathrm{body}\ \mathrm{weight}}\ast 100\%$) were also calculated. The remaining 12 rats were observed for another 14 days (the recovery period).

#### Blood biochemistry measurements

Serum samples, separated from the blood collected on the 7th, 14th, 21st, and 28th days, were analysed with an AU2700 automatic biochemical analyser for the following biochemical indicators: liver function indices (levels of aspartate aminotransferase (AST), alanine aminotransferase (ALT), and alkaline phosphatase (ALP)), kidney function indices (urea nitrogen (BUN), creatinine (CRE)), blood lipid indices (total cholesterol (TC), triglycerides (TGs), high-density lipoprotein (HDL), low-density lipoprotein (LDL)), total protein (TP), albumin (Alb), blood glucose (Glu), total bilirubin (TBIL), creatine phosphokinase (CK)), and blood ion levels (sodium ions (Na^+^), potassium ions (K^+^) and chloride ions (Cl^−^)).

#### Haematological examination

Blood samples collected on the last day of dosing and in the recovery period were analysed with a MEK-6318 K Automatic blood cell analyser for the following haematological indices: red blood cell count (RBC), haemoglobin (HGB), haematocrit (HCT), mean red blood cell volume (MCV), mean red blood cell haemoglobin (MCH), mean red blood cell haemoglobin concentration (MCHC), reticulocyte count (RET), white blood cell count (WBC) and classification (MONO%, LYMPH%, BASO%, EO%, NEUT%), platelet count (PLT) and prothrombin time (PT).

#### Histological observations

After sacrifice, the following main organs were fixed in formalin and made into pathological sections: brain, pituitary, thyroid, gastrointestinal tract, mesenteric lymph nodes, heart, liver, kidney, spleen, lung, testis, epididymis, ovary, and uterus. All sections were stained with haematoxylin and eosin (H&E) for microscopic examination.

#### Safety evaluation

The benchmark dose (BMD) and benchmark dose lower confidence limits (BMDLs) were calculated to assess the safety of TAZF. Toxic symptoms and indices or sensitive changes were regarded as the response. BMD analysis was performed with the Bayesian BMD platform (https://benchmarkdose.com/) [18] and the benchmark response (BMR) was set to 0.1.

### Toxicokinetics test in the satellite groups

#### Animals in each satellite group

SD rats (5 ~ 6 weeks, 3/group/sex) were randomly allocated to three treatment groups and given the same dosage as those in the repeated toxicity experiment for 28 days. On the 1st and 28th days, blood samples were collected at 0.25, 0.5, 1, 2, 4, 6, 10 and 24 h after administration. Eight components in TAZF were detected: berberine, coptisine, epiberberine, palmatine, jatrorrhizine, columbamine, evodiamine and rutaecarpine. Carbamazepine was chosen as the internal standard (IS).

#### Plasma sample preparation

The blood samples were centrifuged to obtain plasma. Then, 300 μL of acetonitrile containing 40 ng of IS was added to 100 μL of plasma. After vortexing for 1 min, the mixture was centrifuged at 17,000 rpm for 15 min at 4 °C. The supernatant of each sample was injected into a UPLC–MS/MS system for determination. The injection volume was 1 μL.

#### UPLC–MS analysis

UPLC–MS analysis was performed on a Thermo Fisher Scientific Ultimate 3000 UPLC system (San Jose, USA) and an API 4000 QTRAP mass spectrometer (Foster City, USA) equipped with an ESI source. The mobile phase consisted of 2 solvents: (A) water containing 0.1% formic acid and (B) 100% acetonitrile. The elution gradient of the mobile phase is shown in Table 2. For MS, multiple reaction monitoring (MRM) in positive ion mode was used as the scanning mode for the main components and IS in the plasma samples. The dwell time (DT), collision energy (CE), quadrupole 1 pre-rod bias (Q1) and quadrupole 3 pre-rod bias (Q3) are listed in Table 3. The other parameters were optimized as follows: the curtain gas was 10 psi, ion source gas 1 and gas 2 were both 55 psi, the ion spray voltage was 5500 V, and the interface temperature was 600 °C.

**Table 2:** The gradient of mobile phase A and B in UPLC-MS

Time (min)	Flow (mL/min)	Phase A (%)	Phase B (%)
Initial	0.3	80	20
2	0.3	60	40
3	0.3	50	50
5	0.3	10	90
5.1	0.3	80	20
8	0.3	80	20

**Table 3:** Acquisition parameters in MRM mode

	Precursor ion Q1 (Da)	Product ion Q3 (Da)	Declustering potential (V)	Collision energy (V)
Carbamazepine	237.1	194.1	80	30
Berberine	336.1	320.1	60	45
Coptisine	320.1	292.1	70	45
Epiberberine	336.1	320.1	60	50
Palmatine	352.1	336.1	60	45
Jatrorrhizine	338.1	322.1	60	45
Columbamine	338.1	322.1	60	45
Evodiamine	304.1	134.1	80	30
rutaecarpine	288.1	273.1	60	50

#### Analytical method validation

Standard curves for berberine were constructed with various concentrations: 0.5, 1, 2, 4, 10, 20, 50, 100, 200 and 400 ng/ml. The concentrations of the other components were 0.5, 1, 2, 4, 5, 7.5, 10, 25, 50 and 100 ng/ml due to their lower concentrations in plasma. The regression coefficient (r) of each equation was calculated, and the values should be over 0.99.

Recovery was assessed by analysing quality control (QC) samples at three concentrations: 1.5, 100 and 300 ng/ml for berberine and 1.5, 25 and 75 ng/ml for the other compounds. Recovery was determined by the following 2 equations, and RE% should be within 20%.

$$\mathrm{recovery}\ \mathrm{rate}=\frac{\mathrm{Measured}\ \mathrm{value}}{\mathrm{QC}\ \mathrm{concentration}}\times 100\%$$

$$\mathrm{RE}\%=\frac{\left|\mathrm{Measured}\ \mathrm{value}-\mathrm{QC}\ \mathrm{concentration}\right|}{\mathrm{QC}\ \mathrm{concentration}}\times 100\%$$

#### Toxicokinetic data analysis

The toxicokinetic parameters were calculated by PKSolver software [19], and the AUC_0-t_ values after 28 days of repeated administration were used to evaluate the internal exposure.

### Associations between internal exposure to the components in TAZF and changes in rat body weight

Linear mixed effect models were applied to explore the relationship between internal exposure to the components and changes in the body weights (BWs) of the rats. The models of internal exposure and changes in BW can be described as follows:

**Table:** 

Basic model:	log(BW) = gender + weight_0_ + days + (1| days)	(a)
Total model:	log(BW) = gender + weight_0_ + days + component1 + (component1| days) + component2 + (component2| days) + …	(b)
Specific model:	log(BW) = gender + weight_0_ + days + component + (component| days)	(c)

where “ log(BW) ” represents the logarithm of the BWs of the rats in each satellite group; “ gender ”, “ weight_0_ ” and “ days ” represent the gender, basic BW and days of treatment, respectively, “ component ”, “ component1 ”, “ component2 ”, etc., represent the internal exposure to the components in plasma (berberine, epiberberine, etc.), and “ (1| days) ” and “ (component| days) ” represent the random effects in the models. The Akaike information criterion (AIC) values from the models were compared. When the AIC values of the component were notably lower than those of the basic model, this component was considered to influence BW changes.

### Network pharmacology analysis

Network pharmacology analysis was performed to investigate the potential toxic mechanisms of TAZF. The components detected in plasma were searched in the Comparative Toxicogenomics Database (CTD, http://ctdbase.org/) for related target genes. Studies have indicated that drug-induced paralytic ileus might cause abdominal bloating [20]. Therefore, “Ileus” and “intestinal obstruction” were chosen as disease/toxic effect keywords, and the related genes in the DisGeNET database (https://www.disgenet.org/) and MalaCards database (https://www.malacards.org/) were gathered. Genes that interacted with both components and diseases were selected to perform protein–protein interaction (PPI) network analysis with STRING (http://string-db.org/). Gene Ontology enrichment analysis was performed using the clusterProfiler package in R 4.1.2.

### Statistical analysis

The results are presented as the mean ± S.D.. R software (4.1.2) was applied to perform t tests and ANOVA. Pictures were constructed in GraphPad Prism 7. A value of *P* < 0.05 was considered statistically significant.

## Results

### Repeated toxicity study

#### Clinical observations

Compared with the vehicle control group, no apparent changes were found in rats in the low- or medium-dose groups. In the high-dose group, asthma and lung rales were observed and lasted for 3 to 4 days after treatment with TAZF for approximately 1 week. Some deaths (male: 5/16; female: 7/16) occurred after several days of abdominal distention and an obvious inhibition of BW gain. Anatomical examinations showed that the intestines were full of air, with the accumulation of deep dark mixtures in the terminal caecum. Asthma and lung rales were also observed in rats receiving the high dose on the 7th day of the recovery period. Fortunately, no rats died during this period.

**Fig. 2:**
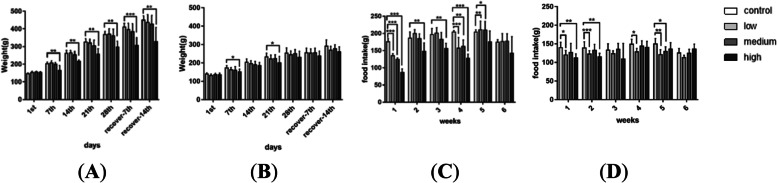
Body weight changes in rats (**A** male, **B** female) and food intake (**C** male, **D** female) during and after oral administration of TAZF for 28 days. Compared with the vehicle control group, *: *P* < 0.05; **: *P* < 0.01; ***: *P* < 0.001

Figure 2 shows the changes in rat BW and food intake. Compared with the vehicle control group, male rats in the high-dose group (Fig. 2A) showed apparent BW gain and food intake reduction from the second week of dosing. The same decline was also observed in females (Fig. 2B). Food intake in the treated groups was also less than that in the vehicle control group (Fig. 2C and D).

#### Organ coefficients

**Fig. 3:**
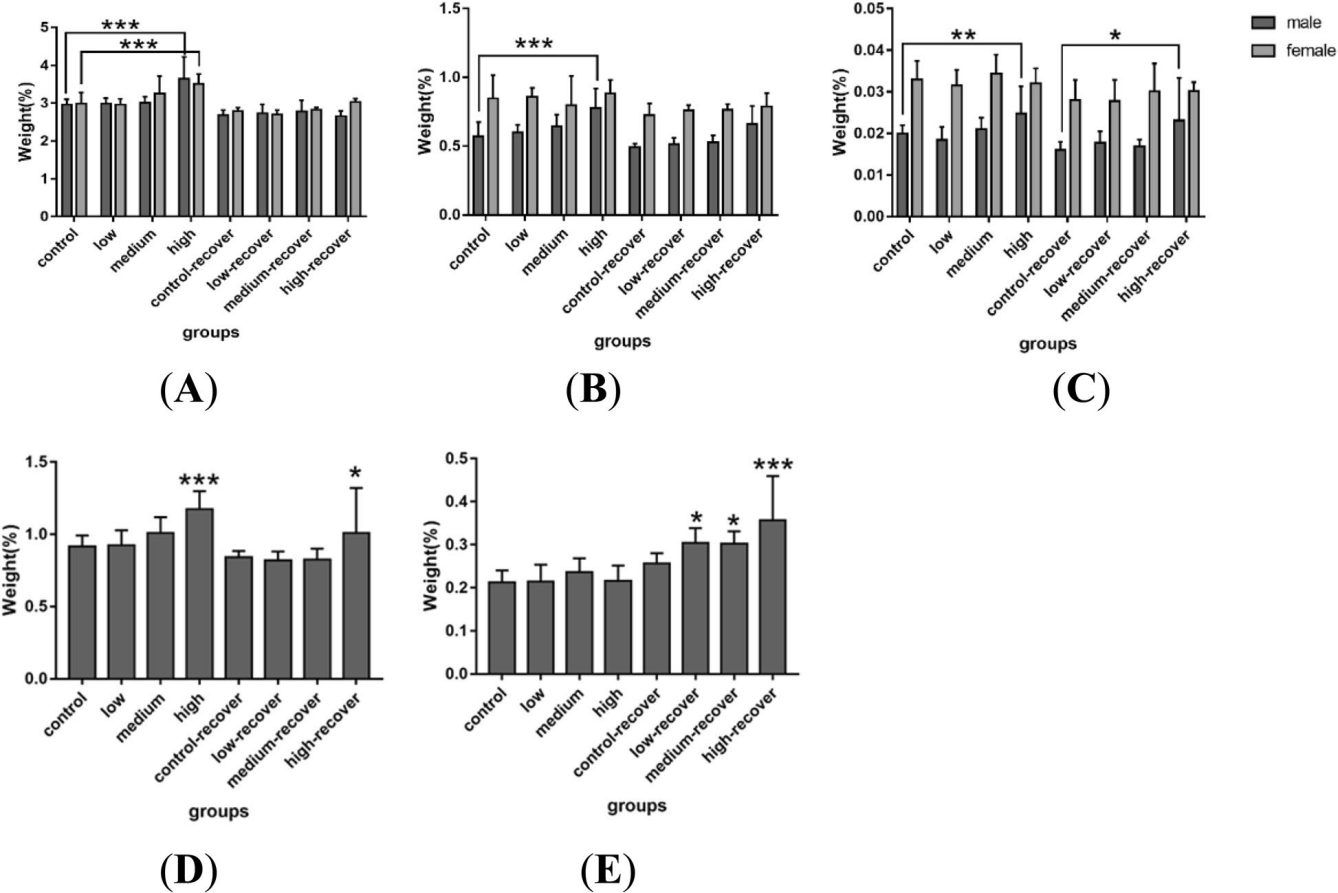
Organ coefficients of the liver (**A**), brain (**B**), adrenal gland (**C**), testis (**D**) and epididymis (**E**) in rats during (*n* = 10) and after (*n* = 6) TAZF administration. Compared with the vehicle control group, *** *P* < 0.001; **: *P* < 0.01; *: *P* < 0.05

Compared with the vehicle control group, the OCs of the kidneys, lungs, spleens, hearts, thymus, ovary, and uterus did not show significant differences (Tables S1 and S2). As shown in Fig. 3A, the liver OCs of the rats in the high-dose group was significantly increased, and this difference disappeared after stopping drug administration. The brain and adrenal gland in the male rats in the high-dose group (Fig. 3B and C) had noticeable weight gain. Male reproductive organs, such as the testis (Fig. 3D) and epididymis (Fig. 3E), were also heavier in the rats in the high-dose group than in those in the control group.

#### Blood biochemistry

**Fig. 4:**
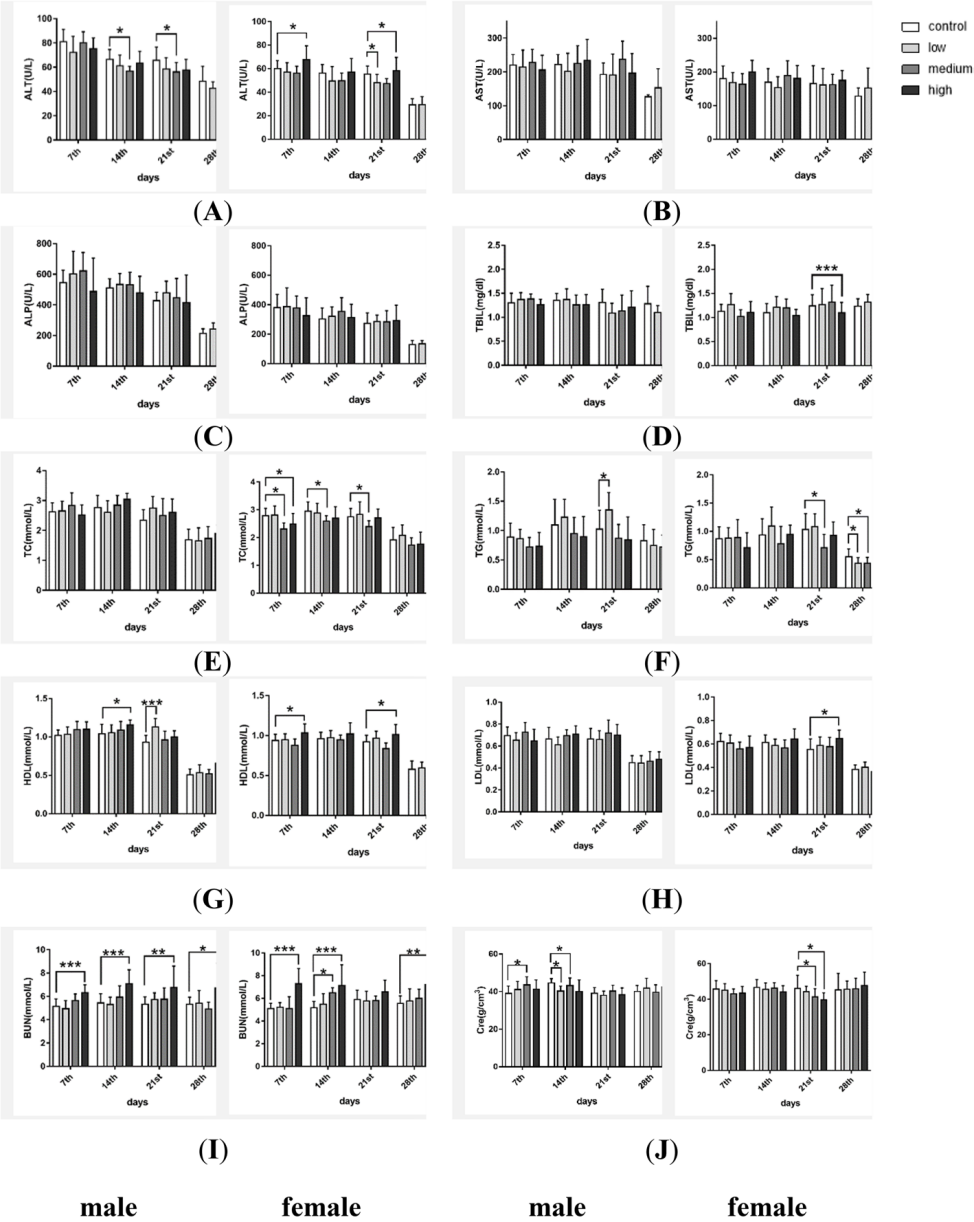
Results of blood biochemistry during TAZF administration. Liver function indices: (**A**) ALT; (**B**) AST; (**C**) ALP; (**D**) TBIL. Blood lipid indices: (**E**) TC; (**F**) TGs; (**G**) HDL; (**H**) LDL. Kidney function indices: (**I**) BUN; (**J**) Cre. Compared with the vehicle control group, ***: *P* < 0.001; **: *P* < 0.01; *: *P* < 0.05

Figure 4 presents the results of blood chemistry during drug administration. Compared with the vehicle control group, liver function indices (ALT, AST, ALP and TBIL levels) did not show significant toxic changes. The decreases in TC and TGs and the increase in HDL started as early as the first week, suggesting a blood lipid decreasing effect. Although BUN and CRE levels increased in several groups, the changes were in the normal reference range for SPF rats [21], which meant that the administration of TAZF was not harmful to the kidney. The results of the plasma protein indices and blood ion levels are presented in Figure S1.

The serum biochemical levels during the recovery period are presented in Figure S2. Most changes, except those of TGs and LDL, which continued to decrease, disappeared after stopping drug administration.

#### Haematological examination

The results of the haematological examination are shown in Tables S3 and S4. Compared with the vehicle control group, the female rats had some changes in RBC and WBC after administration of TAZF. During treatment, the MCV and MCHC in the female rats in the high-dose group increased, while the MCH decreased. The classification of WBCs changed along with an increase in MONO% and a decrease in EO% during the recovery period, indicating that TAZF may affect the distribution of immune cells in peripheral blood.

#### Histological examination

**Fig. 5:**
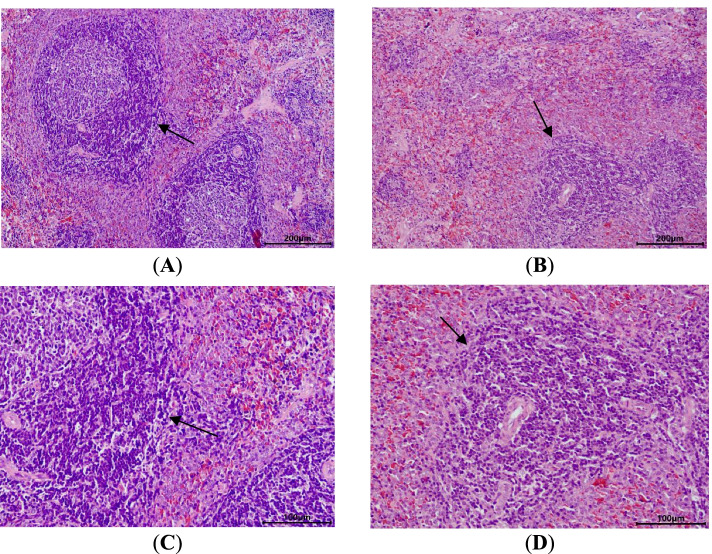
Microphotographs of splenic white pulp atrophy in rats in the high-dose group. (**A**) control group-100×; (**B**) high-dose group-100×; (**C**) control group-200×; (**D**) high-dose group-200×. The border of the splenic white pulp in the control group was clear and the shape was complete, while the splenic white pulp from the rats in the treatment groups had unclear borders with an incomplete shape. The splenic white pulp area and lymphocyte density decreased, and sinus tissue cells proliferated

All of the main organs of the rats in the control and high-dose groups were inspected by histological examination. As shown in Fig. 5, apparent atrophy of splenic white pulp was observed in rats receiving the high dose of TAZF, along with a decrease in lymphocyte density and proliferation of sinus tissue cells. Such changes occurred in 60% of the male (6/10) and 44% of the female rats (4/9) in the high-dose group. No other significant changes were found in the other organs, including the kidney, brain, liver, thymus, lung, male gonad, and digestive tract. These histological changes are presented in Figures S3 and S4.

#### Safety evaluation

BW inhibition was chosen as a measurable toxic index to calculate the benchmark dose of TAZF. As shown in Table 4, the BMDLs of TAZF were 1.27 g/kg for males and 1.91 g/kg for females.

**Table 4:** BMD and BMDL values of body weights changes in rats

Gender	BMD(g/kg)	BMDL(g/kg)
Male	2.27	1.27
Female	3.90	1.91

### Toxicokinetics test

#### Analytical method validation

The linearity regression and linear range results are listed in Table S5. The calibration curves of the components presented satisfactory linearity with r values greater than 0.99. Table S6 presents the sensitivity and recovery results. All the RE% values of the components, except for 1.2 ng/ml evodiamine, showed an acceptable value under 20%, suggesting that these results were accurate.

#### Toxicokinetic profiles

**Fig. 6:**
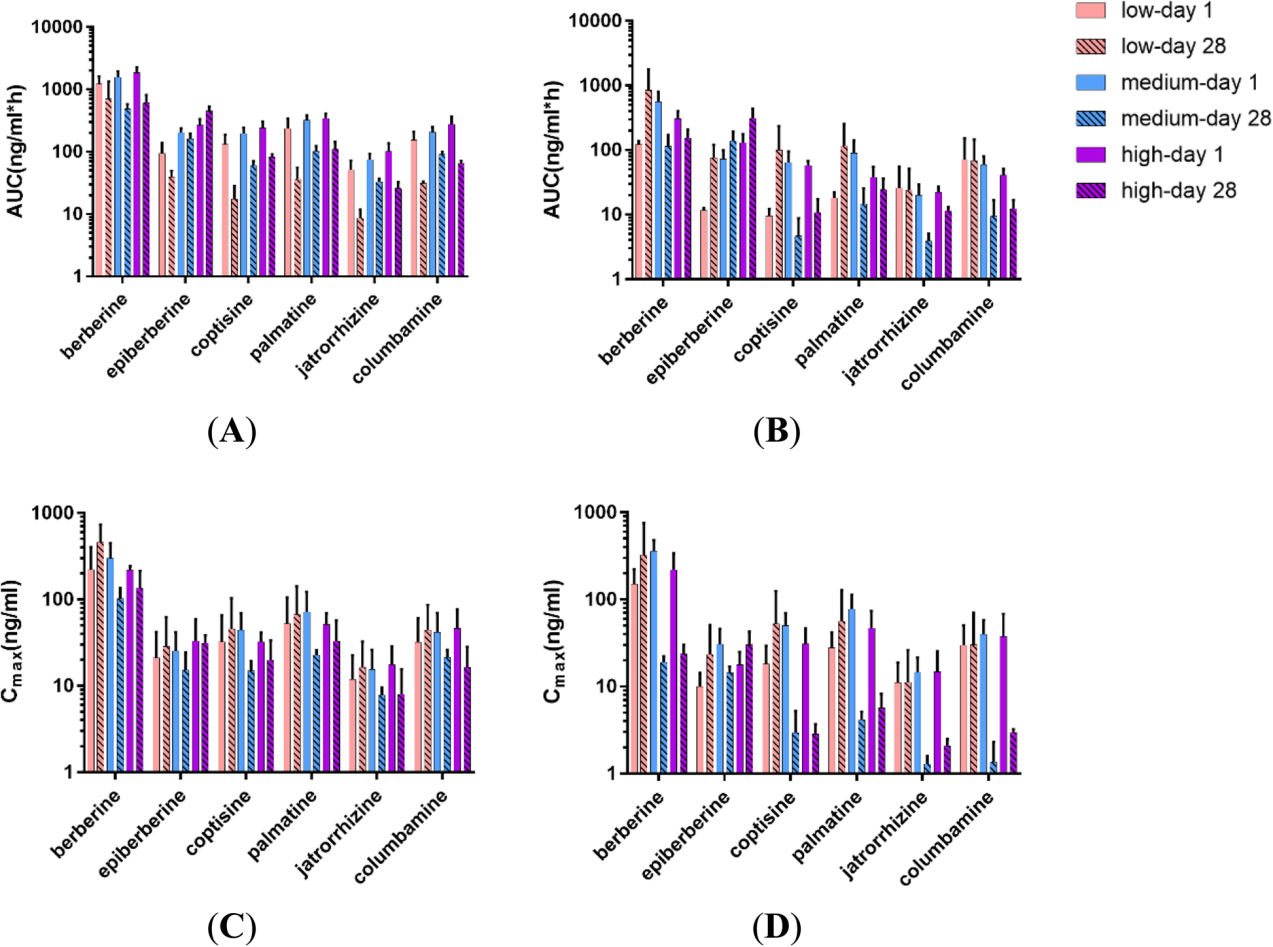
AUC_0-t_((**A**), (**B**)) and C_max_ (ng/ml) ((**C**), (**D**)) of berberine, epiberberine, coptisine, palmatine, jatrorrhizine and columbamine on the first and 28th day of treatment in male ((**A**), (**C**)) and female ((**B**), (**D**)) rats

The levels of evodiamine and rutaecarpine in plasma were under the detection limit (0.5 ng/ml). Figure S5 presents the toxicokinetic profiles of the other six components (berberine, epiberberine, coptisine, palmatine, jatrorrhizine and columbamine) after single and repeated administrations of TAZF. As shown in Fig. 6 and Table 5, after a 28 days of administration, the systemic exposure to berberine, coptisine, palmatine, jatrorrhizine, and columbamine was much lower than that after a single administration. The concentration of berberine in plasma was the highest, suggesting that berberine may play an essential role in the efficacy of TAZF. Most components had higher AUC_0-t_ values in males than in females, and the former also showed significant dose-dependent relationships with the exception of berberine and columbamine on the 28th day. The 28-day AUCs of epiberberine in the high-dose group were 1 to 2 times greater than those of the first day (Fig. 6A and B), indicating that repeated administration might affect the elimination process. The C_max_ of most components increased less than proportionally with the increase in dose. Most components had the highest plasma concentration in the medium-dose group on the first day (Fig. 6C and D). Other toxicokinetic parameters are presented in Table S7.

**Table 5:** Toxicokinetic parameters of 6 components in rat plasma on the first and 28th day treated with TAZF

Components	days	Dose (mg/kg)	C_max_ (ng/ml) (mean ± S.D.)		AUC_0-t_ (ng/ml*h) (mean ± S.D.)	
			male	female	male	female
berberine	Day 1	58.92	221.60 ± 179.72	148.77 ± 72.99	1222.27 ± 389.41	121.57 ± 17.07
		176.76	299.00 ± 148.50	358.33 ± 119.60	1570.11 ± 389.90	554.10 ± 251.73
		545.01	219.33 ± 24.42	218.70 ± 118.35	1860.60 ± 424.79	306.28 ± 94.72
	Day 28	58.92	460.50 ± 275.96	324.10 ± 431.97	699.29 ± 643.96	838.44 ± 949.05
		176.76	102.40 ± 33.71	19.10 ± 3.28	484.04 ± 91.68	115.09 ± 56.44
		545.01	135.93 ± 77.87	23.83 ± 6.25	608.14 ± 203.22	153.27 ± 53.73
epiberberine	Day 1	5.48	21.05 ± 21.05	9.96 ± 4.38	94.67 ± 43.14	11.57 ± 1.08
		16.44	25.37 ± 16.50	30.43 ± 15.40	201.57 ± 35.93	72.49 ± 26.67
		50.69	33.23 ± 25.69	17.80 ± 7.13	265.36 ± 67.81	128.90 ± 48.41
	Day 28	5.48	28.67 ± 33.82	23.34 ± 27.62	38.92 ± 10.07	75.82 ± 44.51
		16.44	15.32 ± 8.98	14.50 ± 2.54	160.77 ± 34.73	138.58 ± 54.58
		50.69	31.27 ± 7.23	30.37 ± 12.39	451.56 ± 79.31	311.25 ± 123.81
coptisine	Day 1	10.08	32.43 ± 33.02	18.11 ± 11.11	131.79 ± 55.97	9.49 ± 2.79
		30.24	44.23 ± 25.22	50.00 ± 19.72	195.05 ± 48.20	63.81 ± 31.66
		93.24	32.30 ± 9.08	31.07 ± 15.51	239.83 ± 65.50	57.11 ± 10.26
	Day 28	10.08	45.49 ± 57.63	52.53 ± 72.46	17.32 ± 11.02	99.93 ± 135.02
		30.24	14.92 ± 4.51	2.95 ± 2.29	60.21 ± 10.20	4.65 ± 4.06
		93.24	20.05 ± 13.57	2.82 ± 0.87	81.65 ± 9.48	10.74 ± 6.60
palmatine	Day 1	12.56	52.47 ± 52.73	27.93 ± 13.97	236.12 ± 103.55	18.08 ± 4.07
		37.68	71.53 ± 51.37	77.60 ± 35.30	318.68 ± 67.35	90.31 ± 50.76
		116.18	51.33 ± 17.52	46.60 ± 27.16	342.51 ± 66.62	38.00 ± 16.92
palmatine	Day 28	12.56	67.20 ± 74.82	55.94 ± 72.88	36.98 ± 18.55	116.47 ± 138.20
		37.68	22.63 ± 3.35	4.16 ± 0.97	102.21 ± 20.79	14.65 ± 10.78
		116.18	32.93 ± 24.17	5.67 ± 2.58	110.18 ± 34.30	24.07 ± 12.21
jatrorrhizine	Day 1	1.88	11.85 ± 10.79	11.04 ± 7.79	51.95 ± 20.41	25.63 ± 30.02
		5.64	15.41 ± 10.60	14.64 ± 6.91	74.52 ± 18.30	19.94 ± 9.16
		17.39	17.66 ± 10.96	14.84 ± 10.66	101.24 ± 35.32	22.12 ± 4.83
	Day 28	1.88	16.40 ± 15.98	11.15 ± 15.03	8.62 ± 3.21	23.94 ± 27.68
		5.64	7.78 ± 1.76	1.29 ± 0.31	33.28 ± 3.59	3.87 ± 1.24
		17.39	7.97 ± 7.64	2.08 ± 0.41	25.64 ± 6.74	11.36 ± 1.79
columbamine	Day 1	3.80	32.13 ± 28.69	29.98 ± 20.67	152.81 ± 54.87	71.44 ± 80.14
		11.40	41.70 ± 28.23	39.63 ± 18.46	206.76 ± 40.77	59.20 ± 20.55
		35.15	46.47 ± 30.26	37.70 ± 30.37	275.76 ± 91.98	41.10 ± 10.03
	Day 28	3.80	44.17 ± 42.31	30.29 ± 40.03	31.11 ± 2.21	68.10 ± 76.87
		11.40	21.37 ± 4.71	1.35 ± 0.97	90.56 ± 9.79	9.42 ± 7.32
		35.15	16.44 ± 11.77	2.98 ± 0.24	65.14 ± 6.74	12.23 ± 4.52

### Relationship between internal exposure to the components and changes in BW

Table 6 shows the AIC values of the linear mixed effect models. Except for the total model, only the model of epiberberine had a lower AIC value than the basic model, indicating that the internal exposure of epiberberine was responsible for the changes in BW in the treated groups. The coefficient of this model was < 0, suggesting that as epiberberine exposure increased, BW increased more slowly.

**Table 6:** AIC values of linear mixed effect models

Models	AIC	coefficient	
Basic model	− 129.42		
Total model	−145.79		***
berberine	− 127.11	0.251	
epiberbeirne	−176.39	−0.001	***
Coptisine	− 134.01	0.102	
Palmatine	−134.69	0.149	
Jatrorrhizine	−140.19	0.271	
Columbamine	−134.59	0.268	

***: *p* value < 0.001

### Network pharmacology analysis

**Fig. 7:**
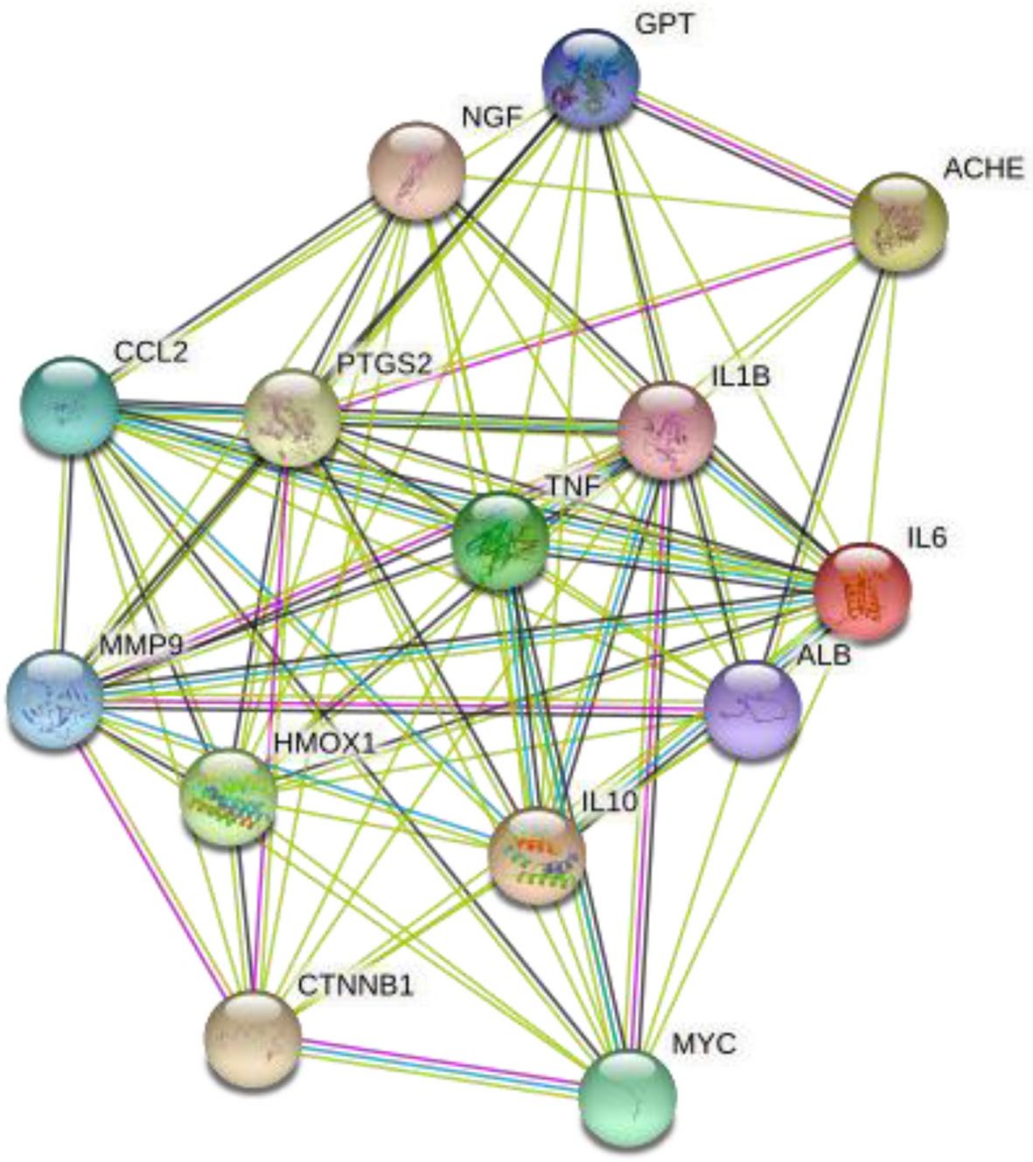
Protein–protein interaction network of target genes from network pharmacology analysis

**Fig. 8:**
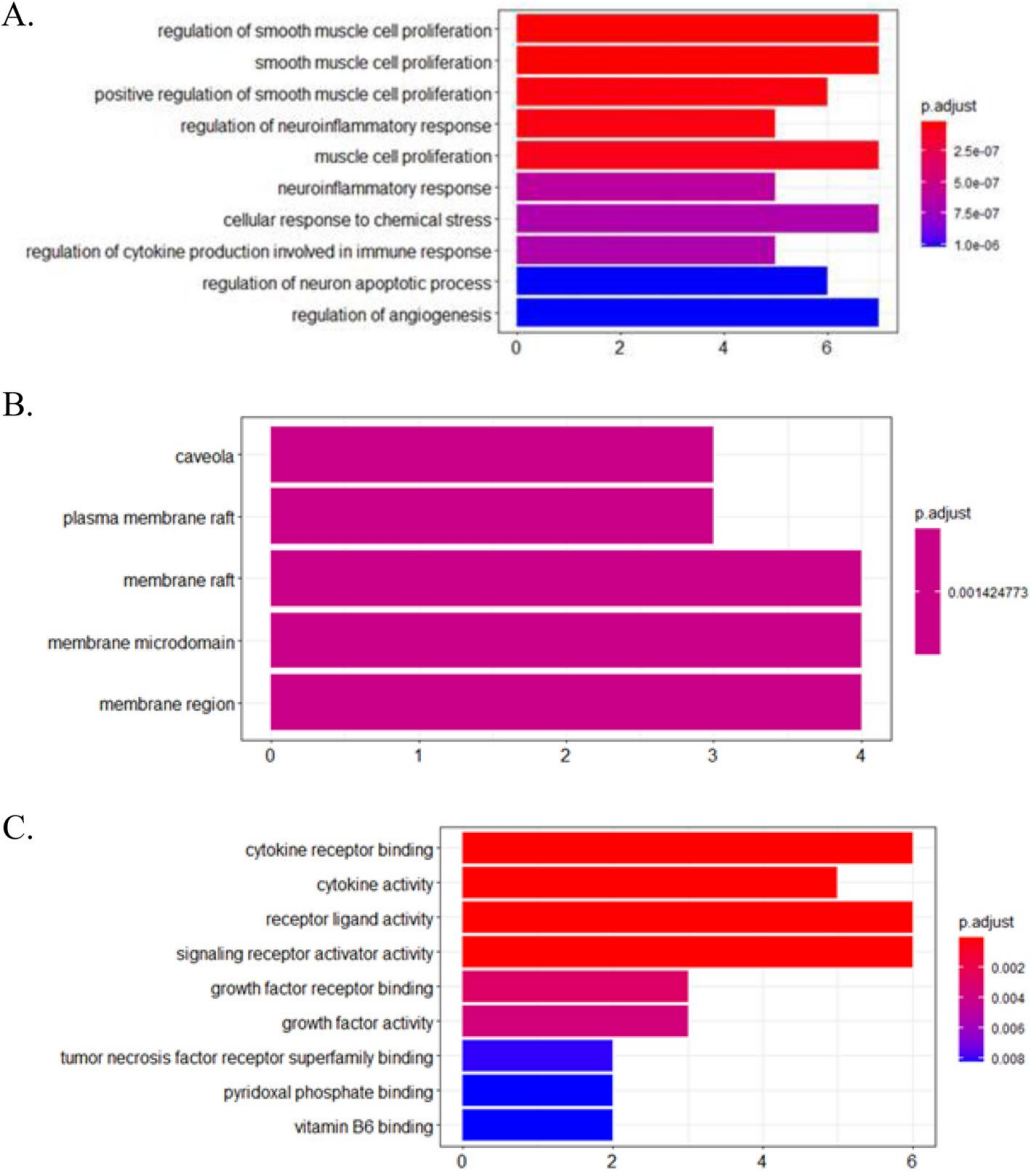
Results of molecular function (**A**), cellular component (**B**) and biological process (**C**) from GO analysis

A total of 271 genes were included in the TAZF component target set, and 136 were included in the ileus-related gene set. Fourteen genes were found in both sets: ACHE, ALB, GPT, NGF, CCL2, HMOX1, IL10, IL1B, IL6, MMP9, PTGS2, TNF, CTNNB1, and MYC. The PPI network shown in Fig. 7 suggests that these genes are related. Figure 8 presents the results of GO analysis. These results showed that target genes might affect smooth muscle cell proliferation and that the cell membrane could be the action site.

## Discussion

In this study, we tried to assess the safety of TAZF via a repeated toxicity experiment. Although TAZF does not show acute toxicity in rats (unpublished data), it can cause a few toxic symptoms after repeated administration. These toxic symptoms appeared in rats in the high-dose (3.7 g/kg) group. The most severe symptom was abdominal bloating, which might lead to death. BW gain inhibition, food intake reduction, and transitory asthma were also observed in our study. Furthermore, atrophy of the splenic white pulp appeared in rats in the high-dose group.

Although *Evodia rutaecarpa* has been regarded as a hepatotoxic ingredient, TAZF treatment did not cause any hepatotoxic symptoms, except an increase in the liver organ coefficient in the high-dose group. The concentrations of evodiamine and rutaecarpine, the potential hepatotoxic components of *Evodia rutaecarpa*, were both low in the plasma. This may explain why TAZF is not hepatotoxic. However, the internal exposure of berberine in this study was much higher than that in other studies [22–26]. A previous study administered pure berberine at a dose of 90 mg/kg to male rats and found that the AUC_0–60_ was 305.8 ng/ml*h, while the C_max_ was 28.2 ng/ml [23]. The AUC_0–24_ and C_max_ in our study were 1222.27 ng/ml*h and 221.6 ng/ml, respectively, when rats were administered TAZF with a dose of berberine of 58.92 mg/kg. Female SD rats were orally administered pure berberine (90 mg/kg), and these AUC_0–36_ and C_max_ values were 88.8 ng/ml*h and 29.2 ng/ml, respectively, while the AUC_0–24_ and C_max_ in our study were 121.57 ng/ml*h and 148.77 ng/ml, respectively, when the female rats were administered 58.92 mg/kg berberine in TAZF. Berberine and other alkaloids, including jatrorrhizine and palmatine, can be metabolized by CYP3A4, CYP1A2 and CYP2D6 [27–30]. These enzymes can also be inhibited by epiberberine and berberine [31–34]. Thus, there might be drug–drug interactions (DDIs) between these alkaloids, which can alter the internal exposure of a single component. More studies are needed to clarify this process.

A sex difference was also observed for the plasma concentrations of the compounds. The gender difference effects were significant regarding the AUCs rather than the C_max_ values [23, 25], indicating that metabolic factors might not be the primary reason for these differences. There were several peaks in the concentration-time profiles, and these multiple peaks played a major role in the sex differences in the pharmacokinetic profiles. Multiple blood concentration peaks from berberine have been reported in male rats [35]. A possible cause of multiple peaks is enterohepatic circulation, as enterohepatic circulation of berberine in rats has been reported [36]. However, no systematic research on enterohepatic circulation and sex differences among these compounds has been performed thus far. Much work needs to be done to investigate the multiple peaks and gender differences.

BW inhibition was chosen as the BMR toxic effect because the blood biochemistry and haematological examination did not exhibit noticeable toxic changes. However, BW can be easily affected by food consumption. Figure 2 reveals the connection between TAZF dose, body weight and food consumption, suggesting that BW could be influenced by a decrease in food consumption. Additionally, BMDLs were calculated based on the dose of TAZF, which contains multiple compounds, and applied to evaluate the safety overall. Although some compounds, such as berberine, exhibited a noticeable difference in exposure (AUC) based on sex, the influence of these compounds on the toxicity of TAZF could be complicated. These might be reasons why the AUC of a single compound showed a sex difference, while the BMDL values did not. Further studies on the relationship between multi-compound exposure and toxic symptoms are needed.

Smooth muscle cells are some of the main cells that make up the digestive tract and are related to peristalsis [20]. Network pharmacology analysis also suggested that these components could affect the proliferation of smooth muscle cells. The effect of berberine on smooth muscle cells has been confirmed by previous studies [37], and may contribute to abdominal bloating symptoms. Berberine is an effective antidiarrhoeal drug [2, 38, 39]. A previous study found that administration of berberine at dosages of 50 and 100 mg/kg for 14 days can improve diarrhoea-predominant irritable bowel syndrome in rats [40]. This effect was also observed in rats with thyroid hormone-induced diarrhoea after 7 days of 60 mg/kg berberine oral gavage [41]. The high-dose TAZF group in the present study received a similar dose of berberine, but this compound was administered for a longer period (28 days), resulting in higher internal exposure to alkaloids. This high level of berberine might contribute to the adverse effects on the intestine found in this study.

The atrophy of splenic white pulp observed in the high-dose TAZF group might also be related to the alkaloids in TAZF. Berberine has been found to have anti-inflammatory activity and immunosuppressive effects [42, 43]. Recent studies showed that mice administered berberine via intraperitoneal injection at a dose of 1 mg/kg had reduced levels of CD^4+^ Th and CD^4+^CXCR^5+^ Tfh cells and an increased level of Foxp^3+^ Tregs [44, 45]. Berberine administered to mice via intraperitoneal injection (10 mg/kg) had a strong immunosuppressive effect and reduced spleen weight in addition to changes in the classification of immune cells in the spleen [46]. These findings may partly explain the atrophy of splenic white pulp observed in our study. Further studies are still needed.

## Conclusion

In the present study, we evaluated the safety of TAZF via 28 days of repeated administration. The BMDL of TAZF was found to be 1.27 g/kg (male) and 1.91 g/kg (female). TAZF has no noticeable liver or kidney toxicity but carries risks of gastrointestinal and immune toxicity at a high dose. Alkaloids from *Coptis chinensis* are the main plasma components related to TAZF’s toxic effects.

## Supplementary Information

**Additional file 1: Figure S1.** Results of plasma protein indexes and blood ion levels during the administration. Compared with vehicle control group ***: *P* < 0.001; **: *P* < 0.01; *: *P* < 0.05. **Figure S2.** Results of blood chemistry after the oral administration of TAZF. Compared with vehicle control group, ***: *P* < 0.001; **: *P* < 0.01; *: *P* < 0.05. **Figure S3.** Microphotographs of liver, kidney and lung in control group and high-dose group. No obvious toxic change was found. Each figure is a representative photomicrograph from a rat in each group. **Figure S4.** Microphotographs of brain and digestive tract in control group and high-dose group. No obvious toxic change was found. Each figure is a representative photomicrograph from a rat in each group. **Figure S5.** Mean plasma concentration profiles of components in rat plasma after 1-day and 28-day oral administration of TAZF. **Table S1.** OCs of lung, spleen and heart in male rats during (*n* = 10) and after (*n* = 6) administration. **Table S2.** OCs of lung, spleen and heart in female rats during (*n* = 10) and after (*n* = 6) administration. **Table S3.** Results of hematological examination in male rats during (*n* = 10) and after (*n* = 6) administration. **Table S4. **Results of haematological examination in female rats during (*n* = 10) and after (*n* = 6) administration. **Table S5.** Linearity and linear range of the components. **Table S6.** Results of sensitivity and recovery. **Table S7.** Pharmacokinetic parameters of 6 components on the first and 28th day treated day in male rats. **Table S8.** Pharmacokinetic parameters of 6 components on the first and 28th day treated day in female rats.

## Data Availability

All data generated or analysed during this study are included in this published article and its supplementary information files.
