# Supplementary material for: Toxicity and toxicokinetics of the ethanol extract of Zuojin formula
Source: BMC Complement Med Ther. 2022 Aug 15;22:220. doi: 10.1186/s12906-022-03684-0 (PMC9377102; doi:10.1186/s12906-022-03684-0)
Supplement: Supplementary file 1 — Additional file 1: Figure S1. Results of plasma protein indexes and blood ion levels during the administration. Compared with vehicle control group ***: P < 0.001; **: P < 0.01; *: P < 0.05. Figure S2. Results of blood chemistry after the oral administration of TAZF. Compared with vehicle control group, ***: P < 0.001; **: P < 0.01; *: P < 0.05. Figure S3. Microphotographs of liver, kidney and lung in control group and high-dose group. No obvious toxic change was found. Each figure is a representative photomicrograph from a rat in each group. Figure S4. Microphotographs of brain and digestive tract in control group and high-dose group. No obvious toxic change was found. Each figure is a representative photomicrograph from a rat in each group. Figure S5. Mean plasma concentration profiles of components in rat plasma after 1-day and 28-day oral administration of TAZF. Table S1. OCs of lung, spleen and heart in male rats during (n = 10) and after (n = 6) administration. Table S2. OCs of lung, spleen and heart in female rats during (n = 10) and after (n = 6) administration. Table S3. Results of hematological examination in male rats during (n = 10) and after (n = 6) administration. Table S4. Results of haematological examination in female rats during (n = 10) and after (n = 6) administration. Table S5. Linearity and linear range of the components. Table S6. Results of sensitivity and recovery. Table S7. Pharmacokinetic parameters of 6 components on the first and 28th day treated day in male rats. Table S8. Pharmacokinetic parameters of 6 components on the first and 28th day treated day in female rats. [file 12906_2022_3684_MOESM1_ESM.docx]

| 1. ALB-male | 1. ALB-female | 1. TP-male | 1. TP-female |  |
| --- | --- | --- | --- | --- |
| 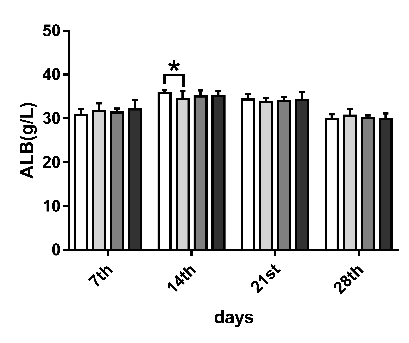 | 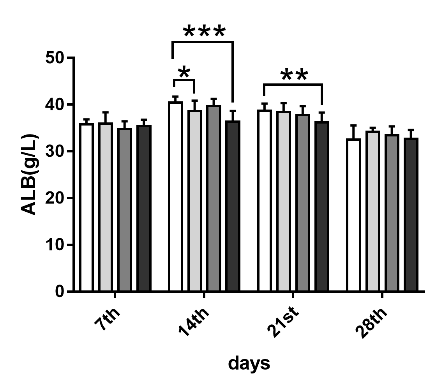 | 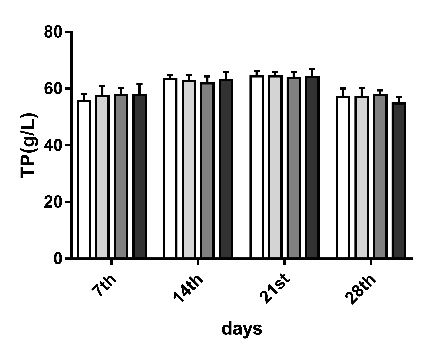 | 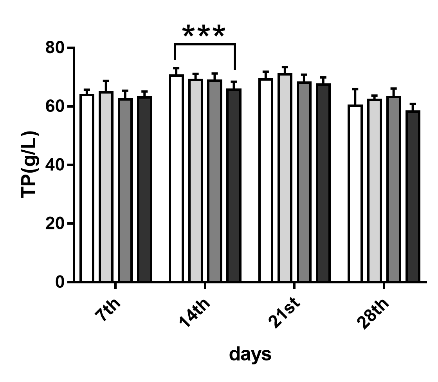 | 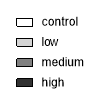 |
| 1. Na^+^-male | 1. Na^+^-female | 1. K^+^-male | 1. K^+^-female |  |
| 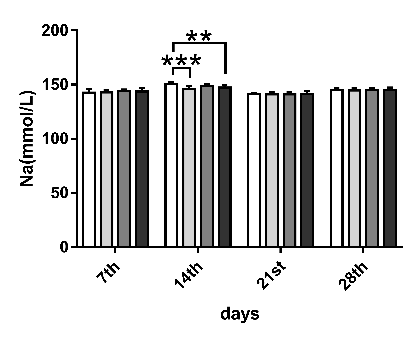 | 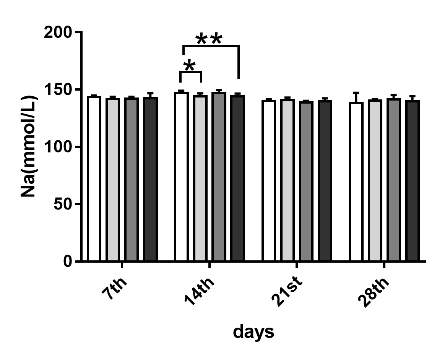 | 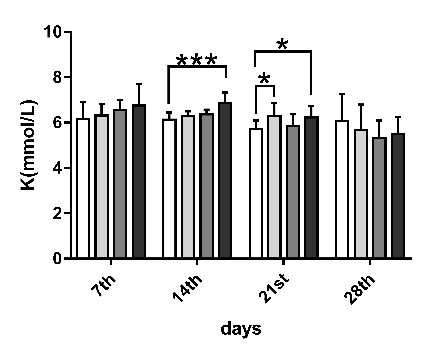 | 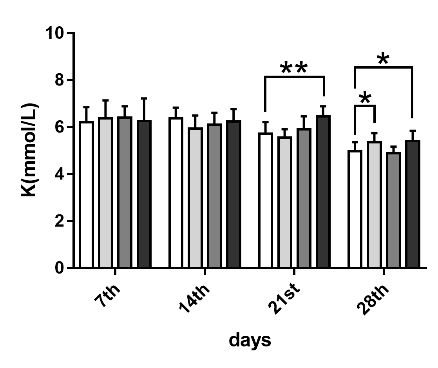 | 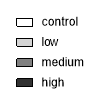 |
| 1. Cl^-^-male | 1. Cl^-^-female |  |  |  |
| 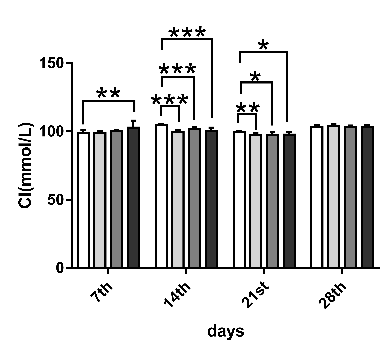 | 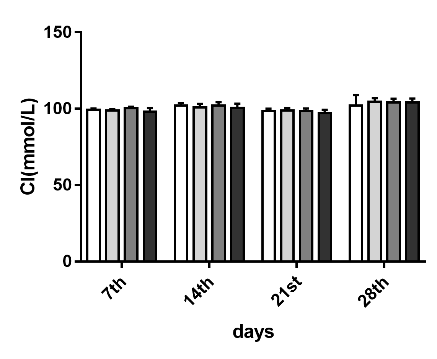 |  |  |  |
| Figure S1 Results of plasma protein indexes and blood ion levels during the administration. Compared with vehicle control group ***: P < 0.001; **: P < 0.01; *: P < 0.05 | | | | |

| **A** | **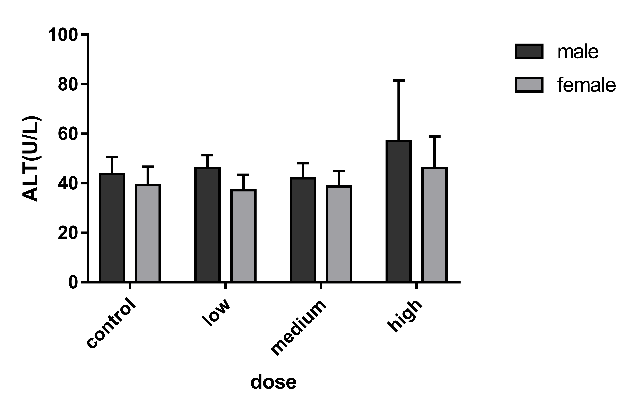** | **B** | **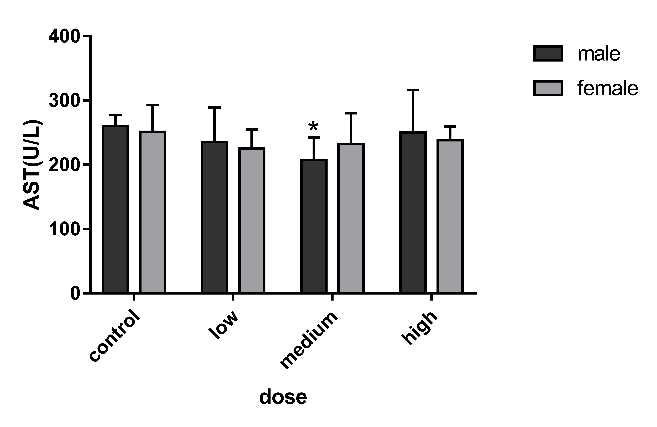** | **C** | **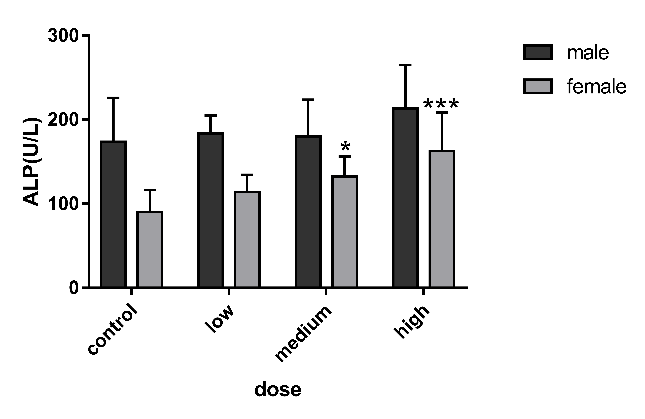** | **D** | **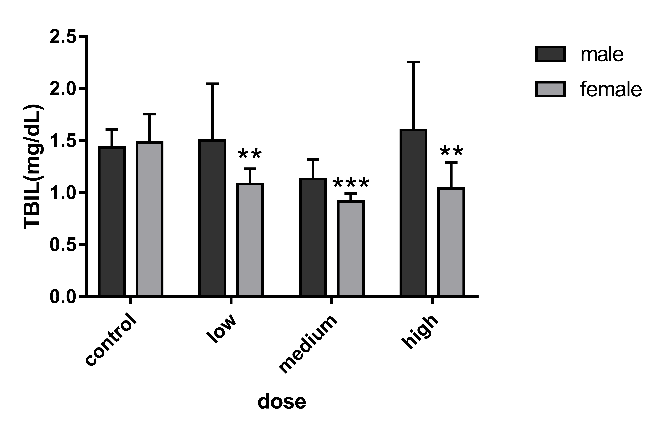** | |
| --- | --- | --- | --- | --- | --- | --- | --- | --- |
| **E** | **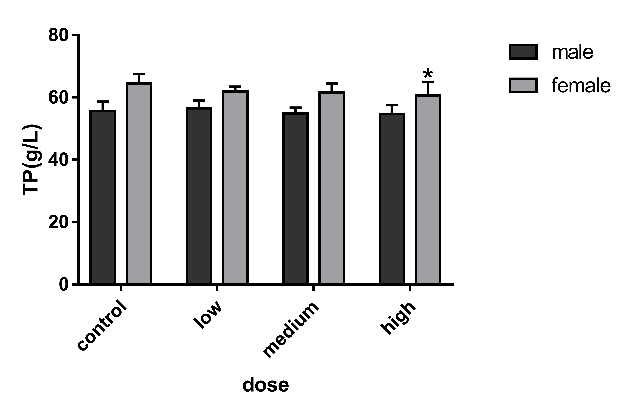** | **F** | **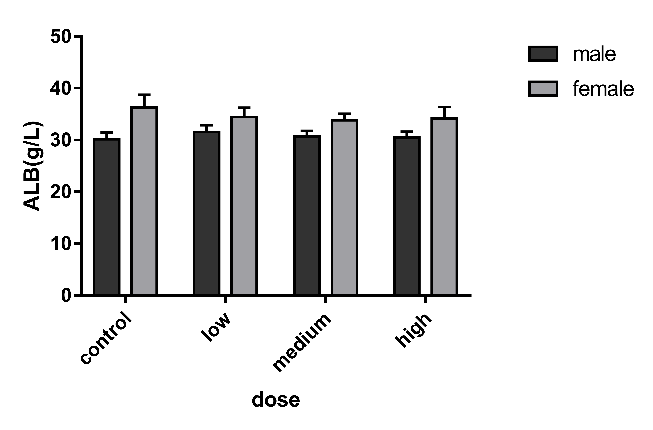** | **G** | **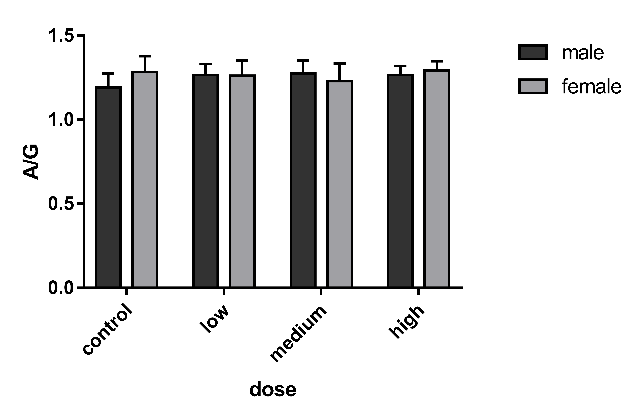** | **H** | **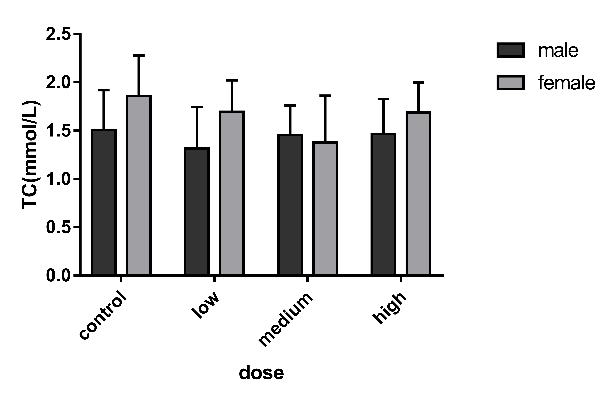** |  |
| **I** | **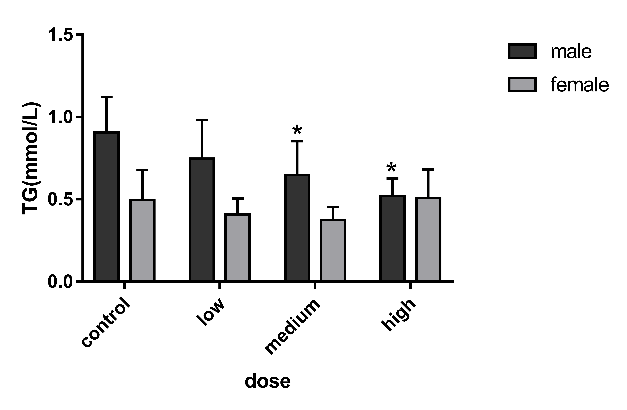** | **J** | **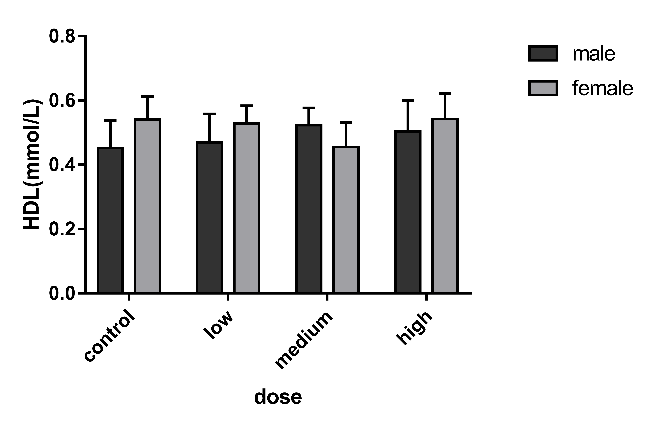** | **K** | **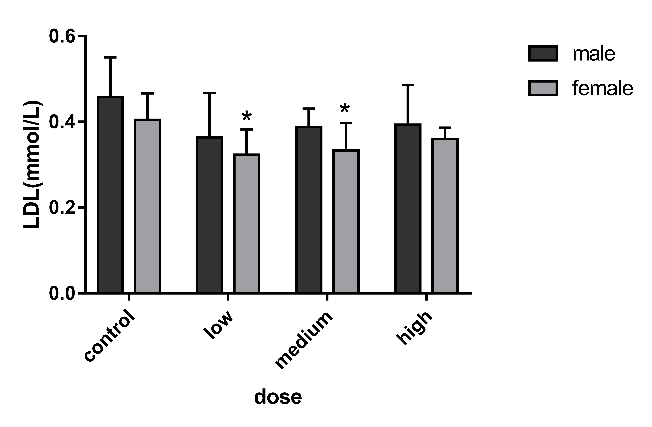** | **L** | **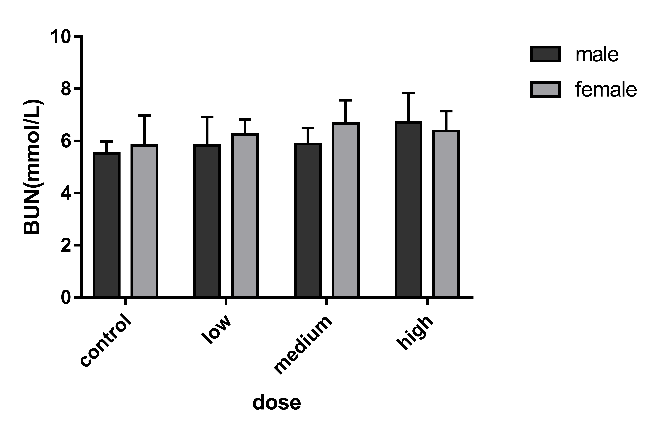** |  |
| **M** | **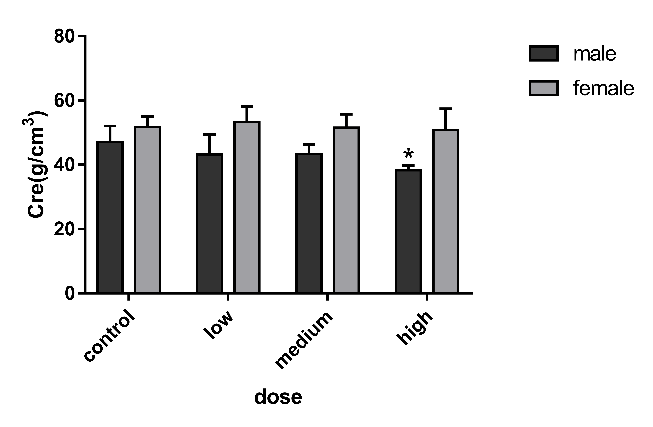** | **N** | **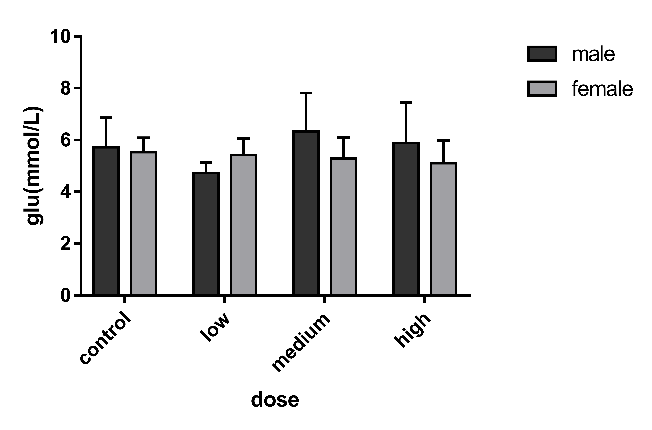** | **O** | **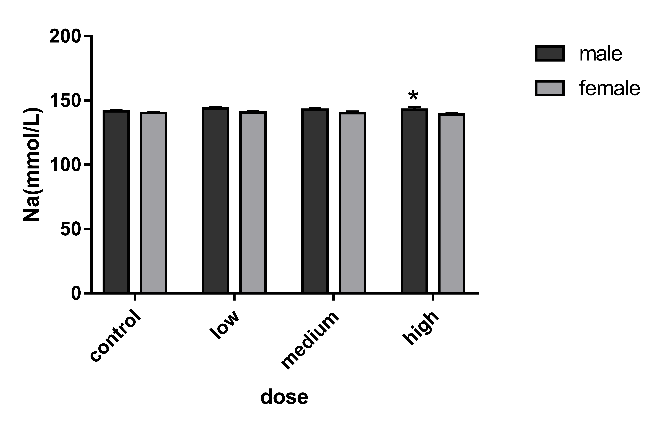** | **P** | **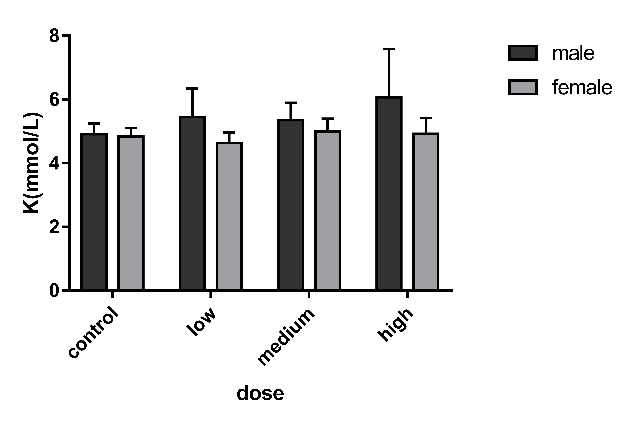** |  |
|  |  | **Q** | **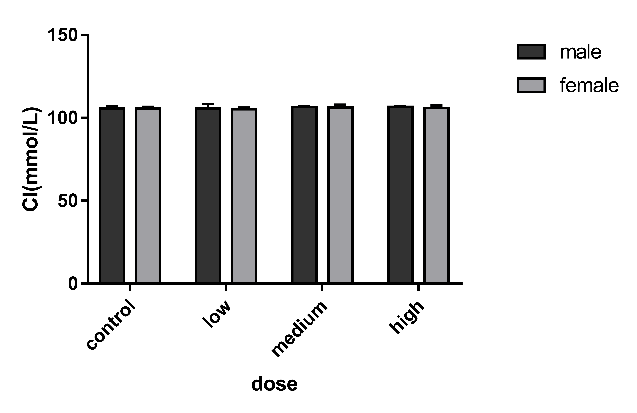** | **R** | **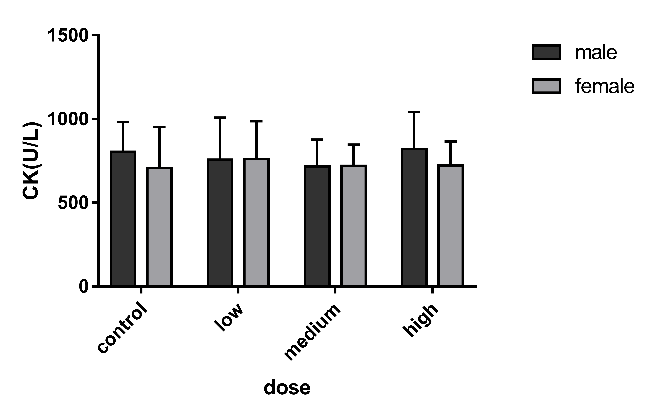** | | |  |
| Figure. S2 Results of blood chemistry after the oral administration of TAZF. Compared with vehicle control group, ***: P < 0.001; **: P < 0.01; *: P < 0.05 | | | | | | | |  |

|  | Control-100x | Control-200x | Sample-100x | Sample-200x |
| --- | --- | --- | --- | --- |
| liver | 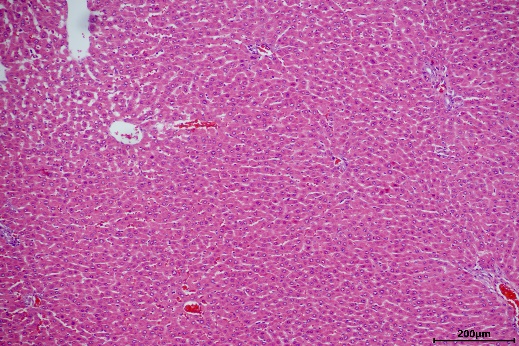 | 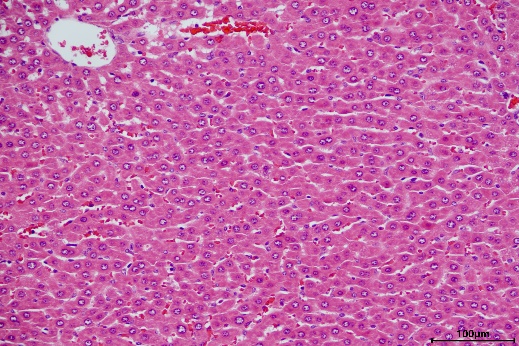 | 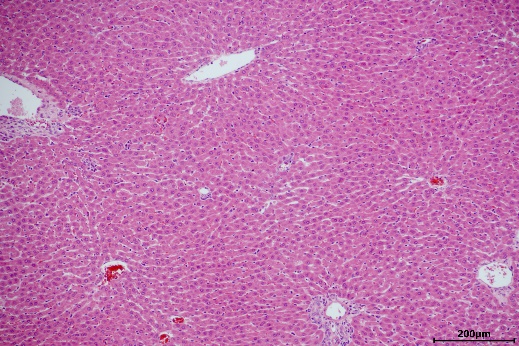 | 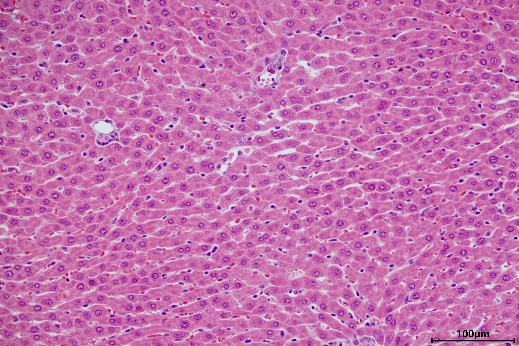 |
| kidney | 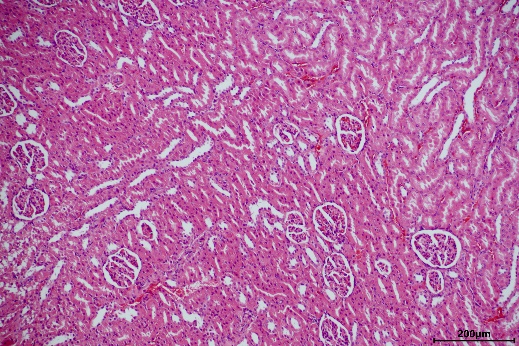 | 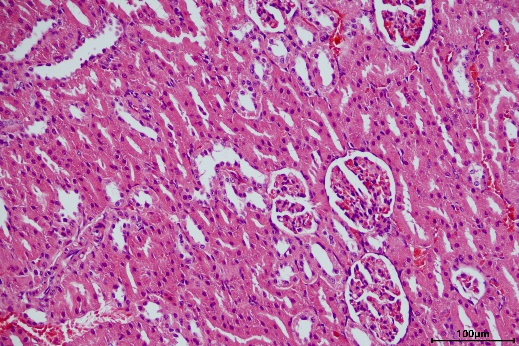 | 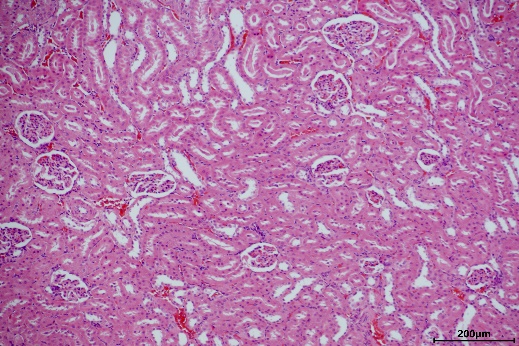 | 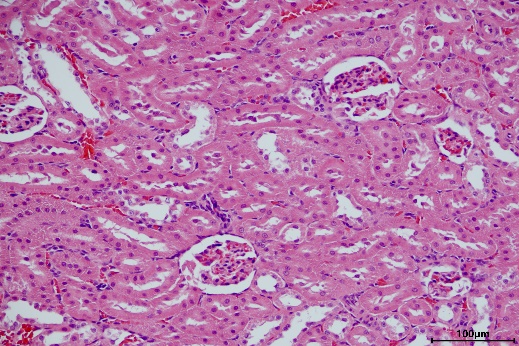 |
| lung | 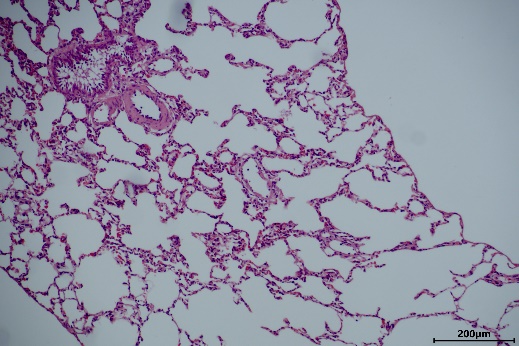 | 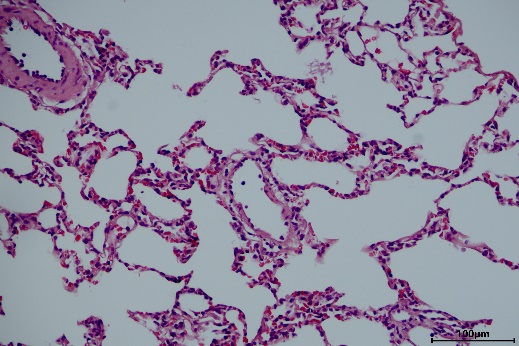 | 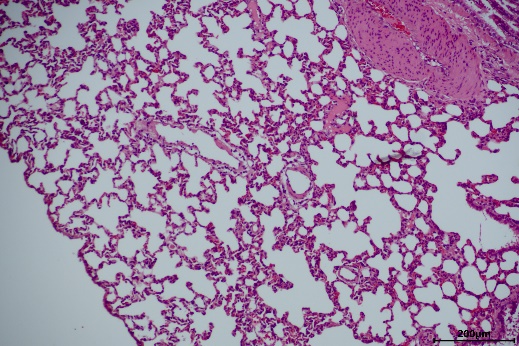 | 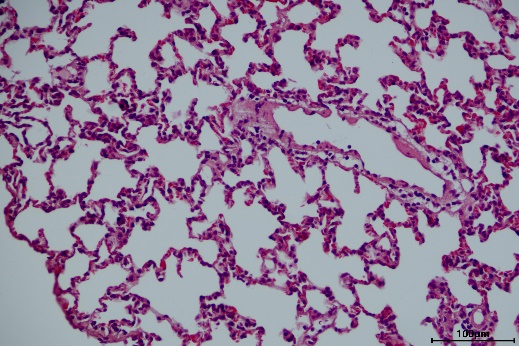 |
| Figure. S3 Microphotographs of liver, kidney and lung in control group and high-dose group. No obvious toxic change was found. Each figure is a representative photomicrograph from a rat in each group. | | | | |

|  |  | Control | Sample |
| --- | --- | --- | --- |
| Brain | 40x | 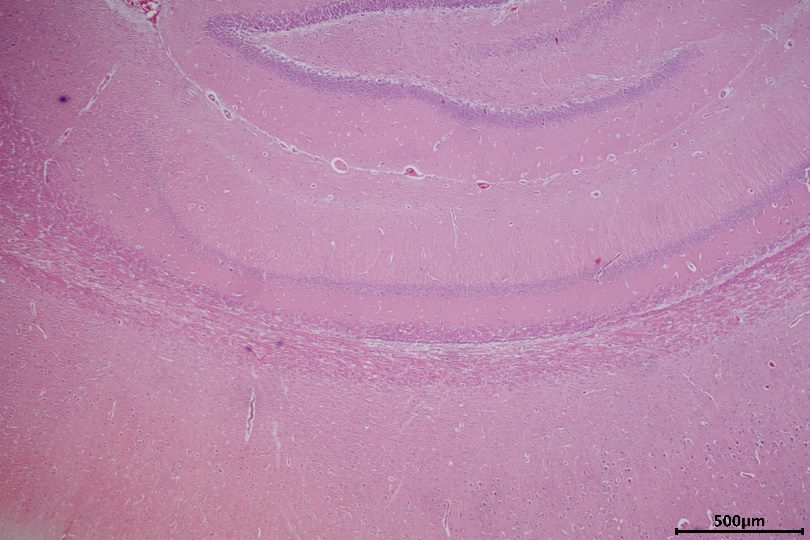 | 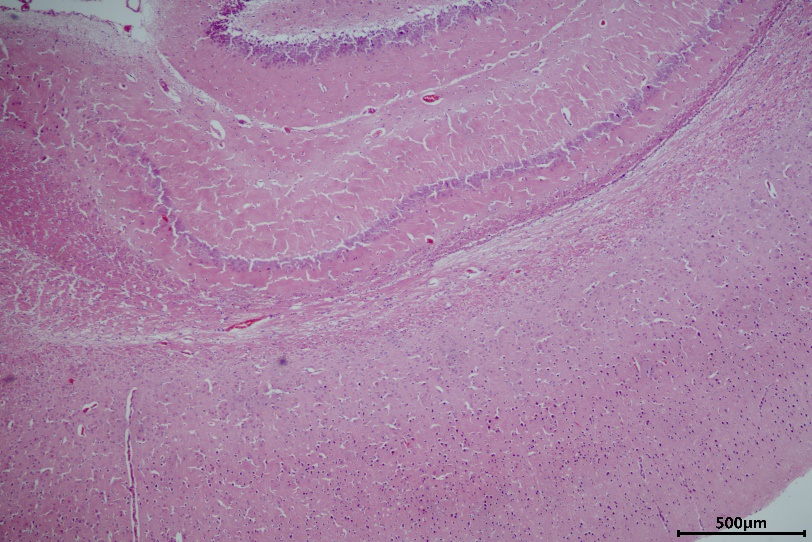 |
| Stomach | 100x | 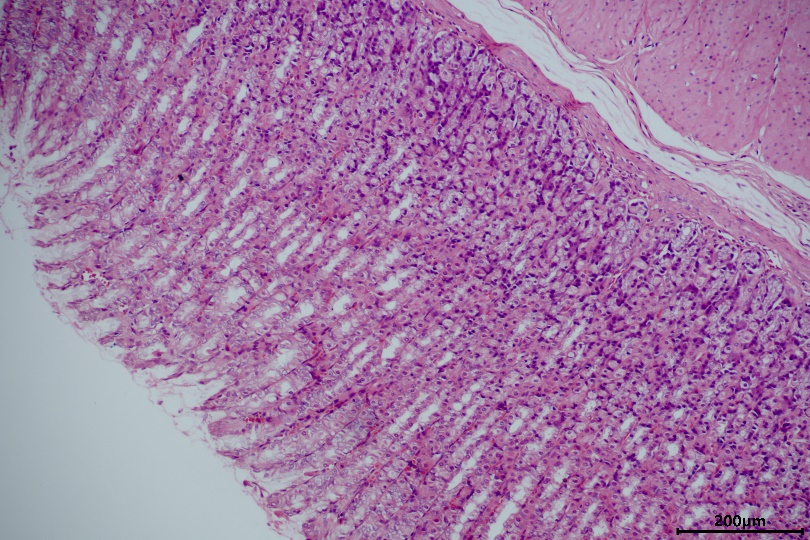 | 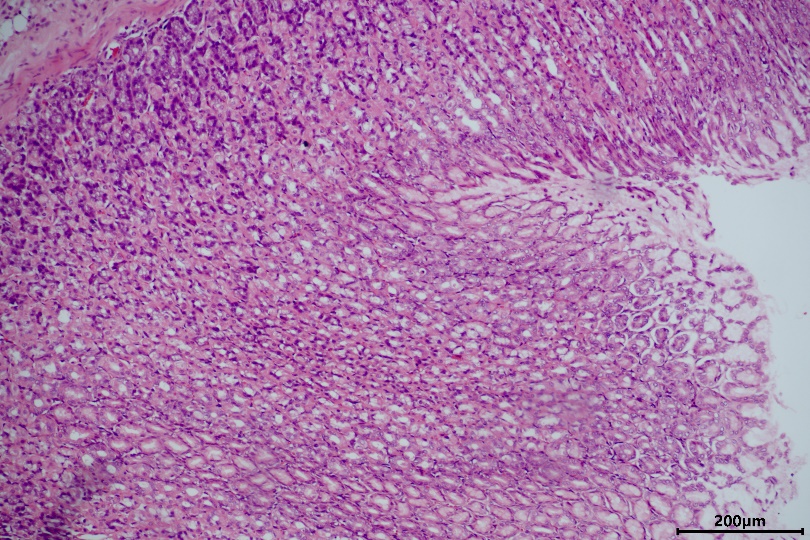 |
| jejunum | 100x | 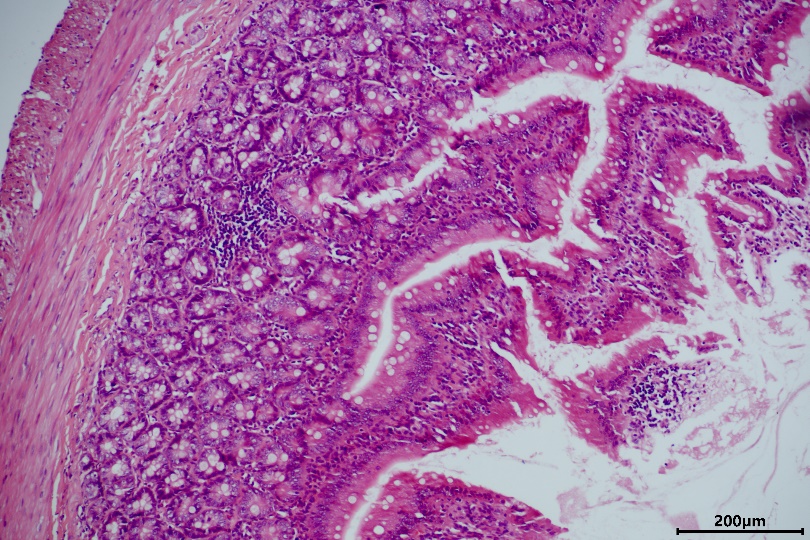 | 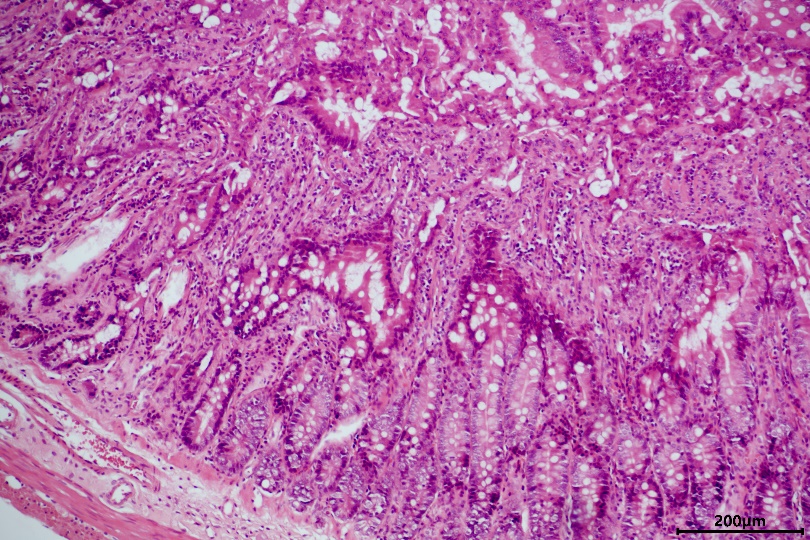 |
| ileum | 100x | 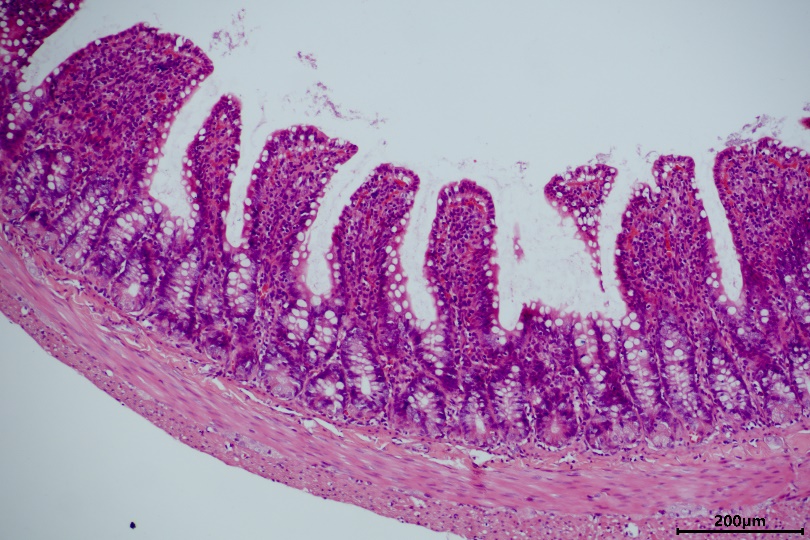 | 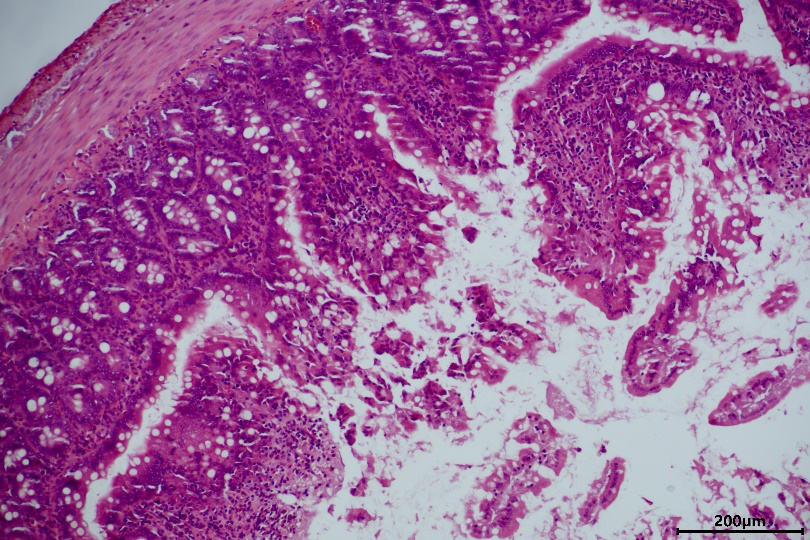 |
| colon | 100x | 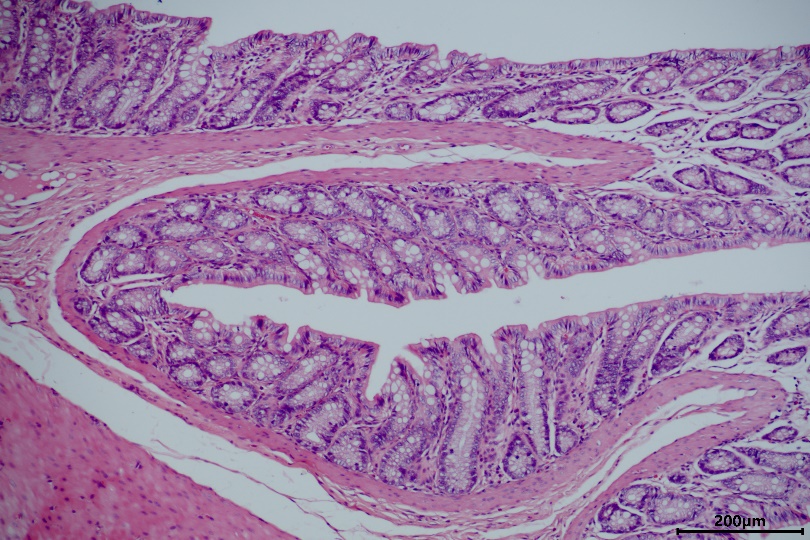 | 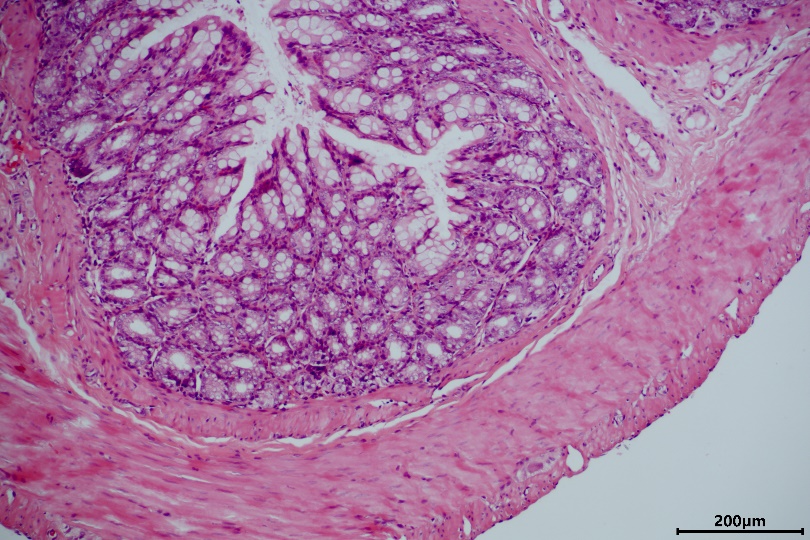 |
| Figure. S4 Microphotographs of brain and digestive tract in control group and high-dose group. No obvious toxic change was found. Each figure is a representative photomicrograph from a rat in each group. | | | |

| 1. Berberine-day 1 | 1. Berberine-day 28 |  |
| --- | --- | --- |
| 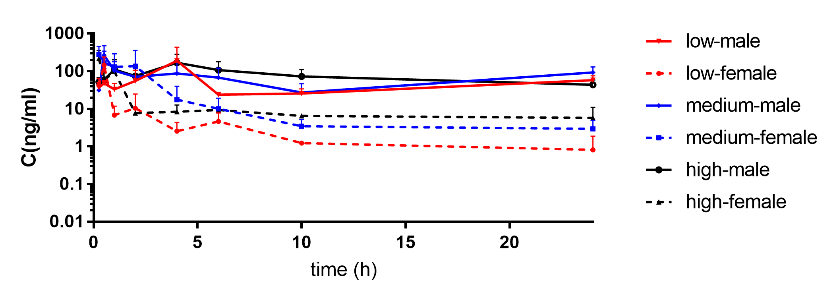 | 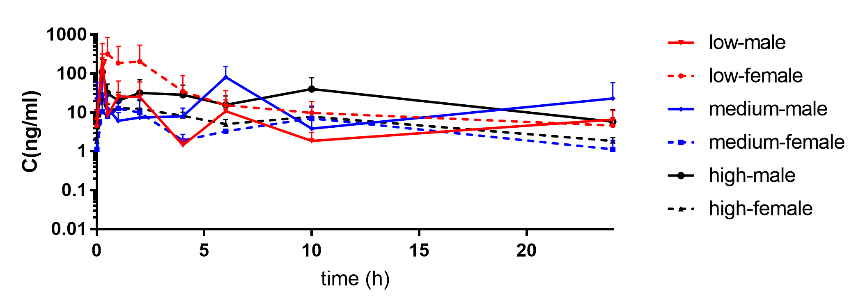 | |
| 1. Epiberberine-day 1 | 1. Epiberberine-day 28 |  |
| 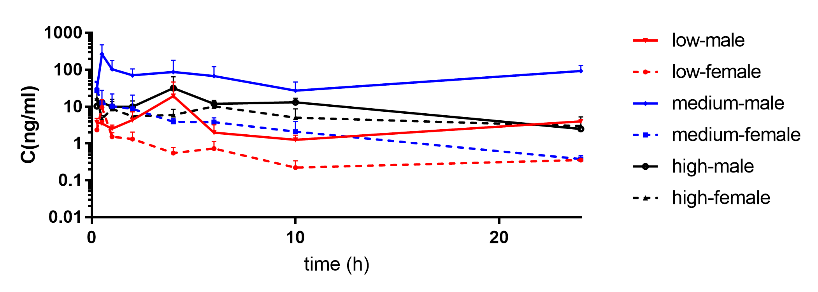 | 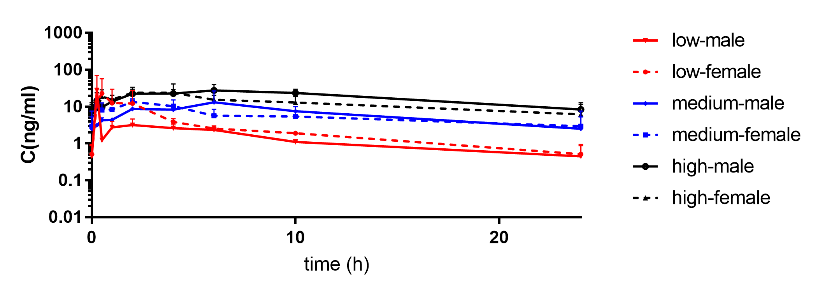 |  |
| 1. Coptisine-day 1 | 1. Coptisine-day 28 |  |
| 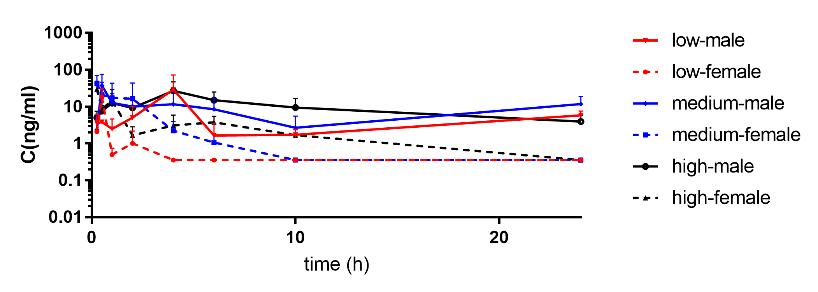 | 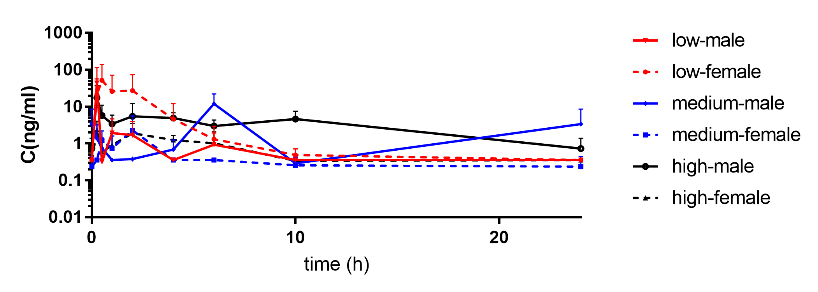 |  |
| 1. Palmatine-day 1 | 1. Palmatine-day 28 |  |
| 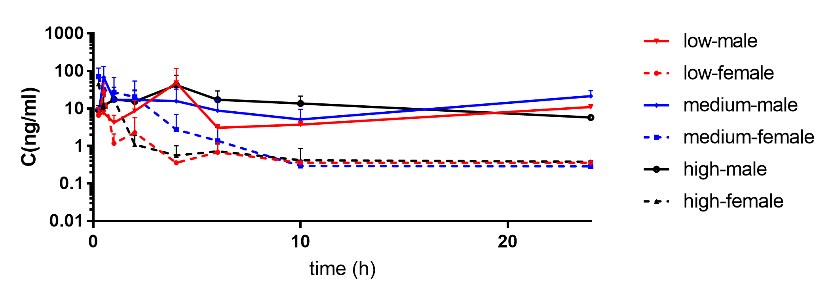 | 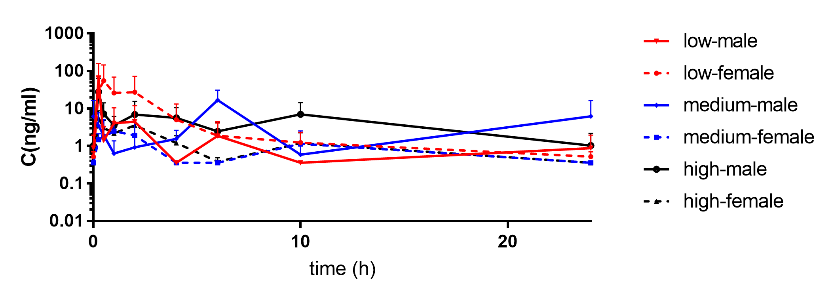 |  |
| 1. Jatrorrhizine-day 1 | 1. Jatrorrhizine-day 28 |  |
| 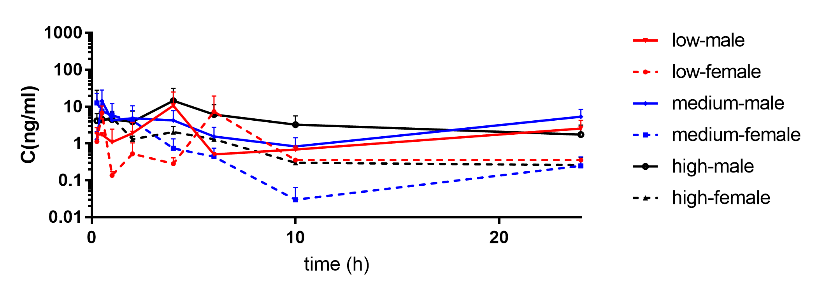 | 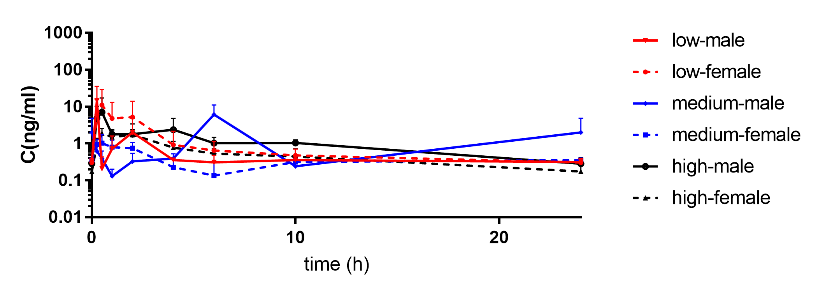 |  |
| 1. Columbamine-day 1 | 1. Columbamine-day 28 |  |
| 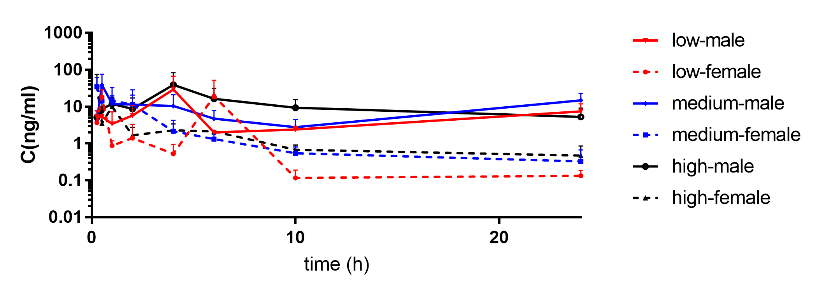 | 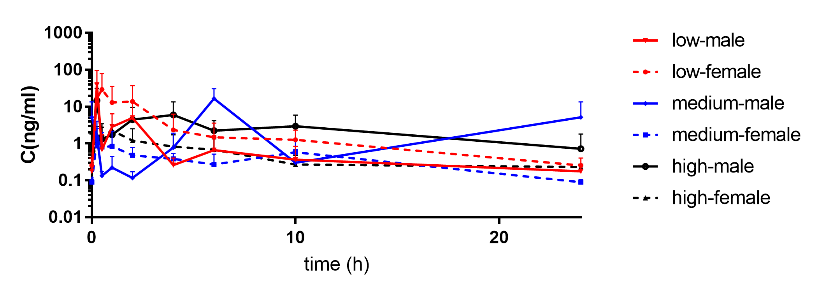 |  |
| Figure. S5 Mean plasma concentration profiles of components in rat plasma after 1-day and 28-day oral administration of TAZF | |  |

Table. S1 OCs of lung, spleen and heart in male rats during (n=10) and after (n=6) administration

| OC | After a 28-day administration | | | | | | | |  | After a 14-day recovery | | | | | | | |
| --- | --- | --- | --- | --- | --- | --- | --- | --- | --- | --- | --- | --- | --- | --- | --- | --- | --- |
|  | control | | low | | medium | | high | |  | control | | low | | medium | | high | |
|  | Mean | S.D. | Mean | S.D. | Mean | S.D. | Mean | S.D. |  | Mean | S.D. | Mean | S.D. | Mean | S.D. | Mean | S.D. |
| lung | 0.41 | 0.072 | 0.42 | 0.063 | 0.42 | 0.039 | 0.46 | 0.088 |  | 0.38 | 0.070 | 0.36 | 0.055 | 0.39 | 0.035 | 0.44 | 0.030 |
| spleen | 0.22 | 0.049 | 0.22 | 0.032 | 0.23 | 0.032 | 0.21 | 0.036 |  | 0.17 | 0.014 | 0.19 | 0.16 | 0.20 | 0.022 | 0.18 | 0.034 |
| heart | 0.36 | 0.032 | 0.35 | 0.032 | 0.38 | 0.098 | 0.36 | 0.033 |  | 0.34 | 0.026 | 0.33 | 0.021 | 0.32 | 0.027 | 0.38 | 0.076 |
| kidney | 0.74 | 0.044 | 0.73 | 0.061 | 0.73 | 0.067 | 0.77 | 0.053 |  | 0.61 | 0.041 | 0.60 | 0.044 | 0.65 | 0.082 | 0.72^**^ | 0.063 |
| thymus | 0.19 | 0.033 | 0.18 | 0.016 | 0.17 | 0.039 | 0.13^*^ | 0.073 |  | 0.17 | 0.112 | 0.11 | 0.021 | 0.13 | 0.018 | 0.13 | 0.038 |
| **: compared with the control group in the same period P < 0.01 *: compared with the control group in the same period P < 0.05 | | | | | | | | | | | | | | | | | |

Table. S2 OCs of lung, spleen and heart in female rats during (n=10) and after (n=6) administration

| OC | After a 28-day administration | | | | | | | |  | After a 14-day recovery | | | | | | | |
| --- | --- | --- | --- | --- | --- | --- | --- | --- | --- | --- | --- | --- | --- | --- | --- | --- | --- |
|  | control | | low | | medium | | high | |  | control | | low | | medium | | high | |
|  | Mean | S.D. | Mean | S.D. | Mean | S.D. | Mean | S.D. |  | Mean | S.D. | Mean | S.D. | Mean | S.D. | Mean | S.D. |
| lung | 0.47 | 0.037 | 0.50 | 0.056 | 0.48 | 0.049 | 0.49 | 0.046 |  | 0.45 | 0.039 | 0.51 | 0.036 | 0.50 | 0.088 | 0.48 | 0.060 |
| spleen | 0.22 | 0.024 | 0.21 | 0.027 | 0.23 | 0.040 | 0.22 | 0.040 |  | 0.20 | 0.018 | 0.22 | 0.012 | 0.28^**^ | 0.16 | 0.21 | 0.037 |
| heart | 0.37 | 0.028 | 0.37 | 0.020 | 0.36 | 0.024 | 0.38 | 0.043 |  | 0.36 | 0.026 | 0.35 | 0.019 | 0.35 | 0.023 | 0.38 | 0.025 |
| kidney | 0.69 | 0.059 | 0.69 | 0.043 | 0.73 | 0.057 | 0.72 | 0.038 |  | 0.63 | 0.032 | 0.62 | 0.047 | 0.65 | 0.043 | 0.66 | 0.034 |
| thymus | 0.21 | 0.040 | 0.22 | 0.036 | 0.21 | 0.035 | 0.21 | 0.058 |  | 0.20 | 0.040 | 0.21 | 0.038 | 0.21 | 0.085 | 0.20 | 0.049 |
| ovary | 0.056 | 0.007 | 0.070 | 0.047 | 0.059 | 0.005 | 0.056 | 0.010 |  | 0.050 | 0.007 | 0.048 | 0.007 | 0.065 | 0.024 | 0.051 | 0.008 |
| uterus | 0.25 | 0.118 | 0.25 | 0.111 | 0.25 | 0.076 | 0.24 | 0.093 |  | 0.22 | 0.103 | 0.21 | 0.116 | 0.20 | 0.087 | 0.19 | 0.017 |
| Compared with the corresponding vehicle control group，** P < 0.01 | | | | | | | | | | | | | | | | | |

Table. S3 Results of hematological examination in male rats during (n=10) and after (n=6) administration

| results | | After a 28-day administration | | | | | | | |  | After a 14-day recovery | | | | | | | | |
| --- | --- | --- | --- | --- | --- | --- | --- | --- | --- | --- | --- | --- | --- | --- | --- | --- | --- | --- | --- |
|  |  | control | | low | | medium | | high | |  | control | | low | | medium | | high | | |
|  |  | Mean | S.D. | Mean | S.D. | Mean | S.D. | Mean | S.D. |  | Mean | S.D. | Mean | S.D. | Mean | S.D. | Mean | S.D. | |
| RBC | M/uL | 7.35 | 0.28 | 7.18 | 0.21 | 7.37 | 0.41 | 7.35 | 0.41 |  | 7.86 | 0.65 | 8.08 | 0.22 | 7.78 | 0.37 | 7.91 | 0.11 | |
| HGB | g/dL | 14.27 | 0.71 | 14.27 | 0.34 | 14.11 | 0.78 | 13.84 | 0.85 |  | 15.03 | 0.74 | 14.67 | 0.77 | 15.03 | 0.53 | 14.60 | 0.17 | |
| HCT | % | 42.98 | 1.72 | 42.41 | 1.50 | 43.20 | 2.57 | 41.96 | 3.09 |  | 43.10 | 2.31 | 42.83 | 1.92 | 43.87 | 1.83 | 42.70 | 1.64 | |
| MCV | fL | 56.78 | 2.42 | 57.51 | 2.23 | 56.75 | 1.88 | 56.36 | 2.37 |  | 54.97 | 2.91 | 53.07 | 3.09 | 56.52 | 3.43 | 54.00 | 2.25 | |
| MCH | pg | 19.40 | 0.97 | 19.74 | 0.79 | 19.40 | 0.69 | 19.11 | 0.66 |  | 19.17 | 0.97 | 18.17 | 1.26 | 19.37 | 1.24 | 18.43 | 0.49 | |
| MCHC | g/dL | 34.12 | 0.33 | 34.27 | 0.71 | 34.16 | 0.29 | 33.94 | 0.68 |  | 34.88 | 0.33 | 34.22* | 0.42 | 34.27 | 0.50 | 34.23 | 1.30 | |
| RET% | % | 5.96 | 1.02 | 6.53 | 2.15 | 5.52 | 1.30 | 5.36 | 2.06 |  | 3.62 | 0.56 | 4.20 | 0.38 | 3.64 | 1.20 | 3.94 | 2.42 | |
| WBC | K/uL | 6.19 | 2.03 | 7.44 | 1.79 | 7.72 | 5.00 | 6.58 | 3.81 |  | 10.62 | 3.09 | 7.51 | 2.22 | 5.88 | 3.43 | 6.91 | 4.10 | |
| NEUT% | % | 10.98 | 5.32 | 15.14 | 4.91 | 12.40 | 6.61 | 16.68 | 10.80 |  | 17.37 | 13.27 | 13.9 | 7.16 | 19.67 | 10.89 | 21.27 | 8.02 | |
| LYMPH% | % | 83.71 | 6.73 | 77.89 | 6.29 | 78.98 | 11.70 | 76.82 | 13.33 |  | 72.03 | 13 | 75.17 | 6.41 | 71.48 | 10.06 | 70.77 | 7.54 | |
| MONO% | % | 4.07 | 1.30 | 5.64 | 1.87 | 7.21 | 8.23 | 5.63 | 2.76 |  | 9.48 | 2.19 | 9.85 | 1.73 | 8.15 | 1.44 | 7.43 | 0.71 | |
| EO% | % | 1.10 | 0.56 | 1.11 | 0.67 | 1.33 | 1.13 | 0.72 | 0.30 |  | 0.97 | 0.21 | 0.95 | 0.59 | 0.55 | 0.30 | 0.40 | 0.36 | |
| BASO% | % | 0.14 | 0.12 | 0.21 | 0.11 | 0.08 | 0.08 | 0.14 | 0.12 |  | 0.15 | 0.05 | 0.13 | 0.08 | 0.15 | 0.20 | 0.13 | 0.15 | |
| PLT | K/uL | 916.10 | 88.43 | 961.71 | 50.97 | 917.80 | 175.68 | 885.33 | 147.51 |  | 811.83 | 74.47 | 832.17 | 91.51 | 852 | 90.13 | 798.67 | 48.39 | |
| PT | s | 12.27 | 0.32 | 12.49 | 0.20 | 12.59 | 0.57 | 12.53 | 0.55 |  | 12.98 | 0.66 | 13.27 | 0.37 | 12.83 | 0.34 | 13.03 | 0.65 | |
| *: compared with the control group in the same period P < 0.05 | | | | | | | | | | | | | | | | | | |  |

Table. S4 Results of haematological examination in female rats during (n=10) and after (n=6) administration

| results | | After a 28-day administration | | | | | | | |  | After a 14-day recovery | | | | | | | |
| --- | --- | --- | --- | --- | --- | --- | --- | --- | --- | --- | --- | --- | --- | --- | --- | --- | --- | --- |
|  |  | control | | low | | medium | | high | |  | control | | low | | medium | | high | |
|  |  | Mean | S.D. | Mean | S.D. | Mean | S.D. | Mean | S.D. |  | Mean | S.D. | Mean | S.D. | Mean | S.D. | Mean | S.D. |
| RBC | M/uL | 7.22 | 0.32 | 7.25 | 0.44 | 7.30 | 0.42 | 7.23 | 0.34 |  | 7.75 | 0.45 | 7.52 | 0.19 | 7.27 | 0.52 | 7.38 | 0.44 |
| HGB | g/dL | 14.30 | 0.65 | 13.99 | 0.80 | 14.24 | 0.82 | 13.29** | 0.72 |  | 14.52 | 0.71 | 15.02 | 0.21 | 14.40 | 0.63 | 14.58 | 0.46 |
| HCT | % | 41.90 | 1.97 | 40.38 | 2.81 | 41.9** | 2.59 | 38.53 | 2.40 |  | 40.93 | 2.14 | 42.25 | 0.74 | 41.02 | 2.00 | 41.14 | 1.48 |
| MCV | fL | 58.78 | 1.75 | 57.83 | 2.93 | 57.96 | 2.55 | 55.50** | 4.20 |  | 52.82 | 0.88 | 56.23* | 1.03 | 56.54* | 2.39 | 55.78 | 1.65 |
| MCH | pg | 19.29 | 0.57 | 19.56 | 0.42 | 19.06 | 1.02 | 18.86 | 0.97 |  | 18.73 | 0.36 | 20.00** | 0.49 | 19.84* | 0.81 | 19.76* | 0.68 |
| MCHC | g/dL | 32.82 | 0.37 | 33.89* | 1.44 | 32.91 | 1.03 | 34.03** | 1.32 |  | 35.47 | 0.62 | 35.55 | 0.37 | 35.12 | 0.29 | 35.44 | 0.52 |
| RET% | % | 6.99 | 1.28 | 6.76 | 0.71 | 6.66 | 0.83 | 6.43 | 1.84 |  | 2.93 | 0.34 | 2.73 | 0.63 | 3.89 | 1.1 | 3.08 | 0.84 |
| WBC | K/uL | 12.68 | 3.24 | 9.57* | 3.33 | 11.88 | 3.51 | 9.82* | 3.83 |  | 5.58 | 1.46 | 6.10 | 0.55 | 6.11 | 1.87 | 5.82 | 1.34 |
| NEUT% | % | 14.25 | 5.38 | 17.45 | 6.20 | 14.42 | 6.20 | 14.44 | 10.38 |  | 21.02 | 5.20 | 14.13 | 7.66 | 23.14 | 17.71 | 22.00 | 5.47 |
| LYMPH% | % | 79.89 | 5.37 | 75.44 | 7.05 | 78.76 | 6.86 | 79.46 | 11.14 |  | 71.37 | 6.10 | 77.97 | 7.07 | 67.82 | 17.36 | 68.72 | 6.75 |
| MONO% | % | 5.14 | 1.26 | 6.28 | 1.55 | 5.99 | 1.88 | 5.41 | 1.53 |  | 6.25 | 0.65 | 6.93 | 1.31 | 8.08* | 0.73 | 8.38* | 1.97 |
| EO% | % | 0.63 | 0.25 | 0.74 | 0.21 | 0.67 | 0.33 | 0.59 | 0.24 |  | 1.12 | 0.40 | 0.83* | 0.23 | 0.72* | 0.32 | 0.76*** | 0.17 |
| BASO% | % | 0.09 | 0.06 | 0.09 | 0.07 | 0.16 | 0.13 | 0.10 | 0.11 |  | 0.25 | 0.18 | 0.13 | 0.10 | 0.24 | 0.05 | 0.14 | 0.09 |
| PLT | K/uL | 922.90 | 89.48 | 834.2 | 193.65 | 880.70 | 142.84 | 924.44 | 117.40 |  | 802.83 | 36.84 | 810.33 | 61.11 | 880.40 | 110.83 | 755.80 | 97.96 |
| PT | s | 12.61 | 0.35 | 12.46 | 0.54 | 12.48 | 0.64 | 12.12 | 0.72 |  | 12.53 | 0.50 | 12.77 | 0.47 | 12.82 | 0.44 | 12.98 | 0.61 |

*: compared with the control group in the same period P < 0.05; **: P < 0.01; ***: P < 0.001

Table.S5 Linearity and linear range of the components

| components | linear range(ng/mL) | Regression Equation | *r* |
| --- | --- | --- | --- |
| berberine | 0.5 - 400 | y = 0.00394x + 0.00845 | 0.9977 |
| epiberberine | 0.5 - 100 | y = 0.0069x + 0.0261 | 0.9978 |
| coptisine | 0.5 - 100 | y = 0.00129x + 0.0271 | 0.9995 |
| palmatine | 0.5 - 100 | y = 0.00608x + 0.0493 | 0.9984 |
| jatrorrhizine | 0.5 - 100 | y = 0.00552x + 0.0131 | 0.9952 |
| columbamine | 0.5 - 100 | y = 0.00208x - 0.000286 | 0.9975 |
| evodiamine | 0.5 - 100 | y = 0.000364x + 0.000611 | 0.9977 |
| rutaecarpine | 0.5 - 100 | y = 0.000473x - 0.00109 | 0.9957 |

Table.S6 Results of sensitivity and recovery

| component | Nominal concentration (ng/ml) | Mean (ng/ml) | RSD | Recovery rate  (%) | RE% |
| --- | --- | --- | --- | --- | --- |
| berberine | 1.5 | 1.4 | 0.21 | 93.3 | 4.66 |
|  | 100 | 80.8 | 0.17 | 80.8 | 19.17 |
|  | 300 | 253.2 | 0.09 | 84.4 | 11.87 |
| epiberberine | 1.5 | 1.2 | 0.15 | 80.0 | 17.22 |
|  | 25 | 22.4 | 0.05 | 89.6 | 10.33 |
|  | 75 | 62.5 | 0.09 | 83.3 | 16.62 |
| coptisine | 1.5 | 1.5 | 0.19 | 100.7 | 0.81 |
|  | 25 | 21.7 | 0.11 | 86.8 | 13.13 |
|  | 75 | 68.5 | 0.09 | 91.3 | 8.73 |
| palmatine | 1.5 | 1.6 | 0.11 | 106.7 | 3.44 |
|  | 25 | 22.0 | 0.07 | 88.0 | 12.00 |
|  | 75 | 64.5 | 0.10 | 86.0 | 14.04 |
| jatrorrhizine | 1.5 | 1.4 | 0.08 | 93.3 | 4.44 |
|  | 25 | 23.0 | 0.16 | 92.0 | 7.87 |
|  | 75 | 60.7 | 0.08 | 80.9 | 19.07 |
| columbamine | 1.5 | 1.7 | 0.13 | 113.3 | 13.44 |
|  | 25 | 23.8 | 0.06 | 95.2 | 4.87 |
|  | 75 | 64.0 | 0.07 | 85.3 | 14.64 |
| evodiamine | 1.5 | 1.2 | 0.22 | 80.0 | 22.08 |
|  | 25 | 20.9 | 0.18 | 83.6 | 16.27 |
|  | 75 | 60.6 | 0.10 | 80.8 | 19.27 |
| rutaecarpine | 1.5 | 1.3 | 0.33 | 86.7 | 16.93 |
|  | 25 | 20.2 | 0.20 | 80.8 | 19.13 |
|  | 75 | 61.3 | 0.13 | 81.7 | 18.33 |

Table. S7 Pharmacokinetic parameters of 6 components on the first and 28^th^ day in male rats.

| components | days | Dose  (mg/kg) | T_1/2_  (h) | | T_max_  (h) | | C_max_  (ng/ml) | | AUC_0-t_  (ng/ml*h) | |
| --- | --- | --- | --- | --- | --- | --- | --- | --- | --- | --- |
|  |  |  | mean | S.D. | mean | S.D. | mean | S.D. | mean | S.D. |
| berberine | Day 1 | 8.01 | 42.28 | 30.18 | 10.00 | 9.93 | 221.60 | 179.72 | 1222.27 | 389.41 |
|  |  | 24.03 | 176.75 | 151.08 | 1.83 | 1.55 | 299.00 | 148.50 | 1570.11 | 389.90 |
|  |  | 72.08 | 13.11 | 1.68 | 3.00 | 1.41 | 219.33 | 24.42 | 1860.60 | 424.79 |
|  | Day 28 | 8.01 | 15.39 | 3.19 | 1.08 | 0.72 | 460.50 | 275.96 | 699.29 | 643.96 |
|  |  | 24.03 | 4.09 | 1.95 | 12.00 | 8.49 | 102.40 | 33.71 | 484.04 | 91.68 |
|  |  | 72.08 | 11.68 | 6.22 | 3.50 | 4.60 | 135.93 | 77.87 | 608.14 | 203.22 |
| epiberberine | Day 1 | 0.82 | 9.40 | 4.43 | 3.33 | 0.94 | 21.05 | 21.05 | 94.67 | 43.14 |
|  |  | 2.47 | 39.62 | 23.38 | 1.67 | 1.65 | 25.37 | 16.50 | 201.57 | 35.93 |
|  |  | 7.42 | 8.06 | 1.73 | 3.33 | 0.94 | 33.23 | 25.69 | 265.36 | 67.81 |
|  | Day 28 | 0.82 | 6.90 | 5.85 | 0.83 | 0.82 | 28.67 | 33.82 | 38.92 | 10.07 |
|  |  | 2.47 | 648.60 | 911.18 | 4.67 | 1.89 | 15.32 | 8.98 | 160.77 | 34.73 |
|  |  | 7.42 | 7.60 | 3.72 | 7.33 | 1.89 | 31.27 | 7.23 | 451.56 | 79.31 |
| coptisine | Day 1 | 1.38 | 13.22 | 6.23 | 3.33 | 0.94 | 32.43 | 33.02 | 131.79 | 55.97 |
|  |  | 2.47 | 31.11 | 16.98 | 1.83 | 1.55 | 44.23 | 25.22 | 195.05 | 48.20 |
|  |  | 7.42 | 8.64 | 0.80 | 3.33 | 0.94 | 32.30 | 9.08 | 239.83 | 65.50 |
|  | Day 28 | 1.38 | 6.11 | 2.88 | 1.08 | 0.72 | 45.49 | 57.63 | 17.32 | 11.02 |
|  |  | 2.47 | 2.60 | 1.24 | 12.00 | 8.49 | 14.92 | 4.51 | 60.21 | 10.20 |
|  |  | 7.42 | 7.81 | 5.59 | 4.08 | 4.24 | 20.05 | 13.57 | 81.65 | 9.48 |
| palmatine | Day 1 | 2.03 | 12.88 | 6.07 | 3.33 | 0.94 | 52.47 | 52.73 | 236.12 | 103.55 |
|  |  | 6.09 | 26.57 | 12.53 | 0.67 | 0.24 | 71.53 | 51.37 | 318.68 | 67.35 |
|  |  | 18.26 | 10.38 | 0.58 | 3.33 | 0.94 | 51.33 | 17.52 | 342.51 | 66.62 |
|  | Day 28 | 2.03 | 12.22 | 6.98 | 0.83 | 0.82 | 67.20 | 74.82 | 36.98 | 18.55 |
|  |  | 6.09 | 2.90 | 1.39 | 12.00 | 8.49 | 22.63 | 3.35 | 102.21 | 20.79 |
|  |  | 18.26 | 11.01 | 8.44 | 4.08 | 4.24 | 32.93 | 24.17 | 110.18 | 34.30 |
| jatrorrhizine | Day 1 | 0.31 | 52.89 | 40.88 | 10.00 | 9.93 | 11.85 | 10.79 | 51.95 | 20.41 |
|  |  | 0.93 | 68.04 | 32.15 | 1.83 | 1.55 | 15.41 | 10.60 | 74.52 | 18.30 |

Table. S7 (Continued)

| components | days | Dose  (mg/kg) | T_1/2_  (h) | | T_max_  (h) | | C_max_  (ng/ml) | | AUC_0-t_  (ng/ml*h) | |
| --- | --- | --- | --- | --- | --- | --- | --- | --- | --- | --- |
|  |  |  | mean | S.D. | mean | S.D. | mean | S.D. | mean | S.D. |
| jatrorrhizine | Day 1 | 2.80 | 9.68 | 3.31 | 4.00 | 1.63 | 17.66 | 10.96 | 101.24 | 35.32 |
|  | Day 28 | 0.31 | 11.10 | 6.50 | 0.83 | 0.82 | 16.40 | 15.98 | 8.62 | 3.21 |
|  |  | 0.93 | 2.72 | 1.29 | 12.00 | 8.49 | 7.78 | 1.76 | 33.28 | 3.59 |
|  |  | 2.80 | 4.48 | 0.81 | 1.17 | 0.62 | 7.97 | 7.64 | 25.64 | 6.74 |
| columbamine | Day 1 | 0.66 | 47.75 | 36.46 | 10.00 | 9.93 | 32.13 | 28.69 | 152.81 | 54.87 |
|  |  | 1.98 | 59.95 | 28.26 | 1.83 | 1.55 | 41.70 | 28.23 | 206.76 | 40.77 |
|  |  | 5.93 | 10.86 | 3.08 | 4.00 | 1.63 | 46.47 | 30.26 | 275.76 | 91.98 |
|  | Day 28 | 0.66 | 7.52 | 2.12 | 0.83 | 0.82 | 44.17 | 42.31 | 31.11 | 2.21 |
|  |  | 1.98 | 4.07 | 1.92 | 12.00 | 8.49 | 21.37 | 4.71 | 90.56 | 9.79 |
|  |  | 5.93 | 11.25 | 8.42 | 4.08 | 4.24 | 16.44 | 11.77 | 65.14 | 6.74 |

Table. S8 Pharmacokinetic parameters of 6 components on the first and 28^th^ day in female rats.

| components | days | Dose  (mg/kg) | T_1/2_  (h) | | T_max_  (h) | | C_max_  (ng/ml) | | AUC_0-t_  (ng/ml*h) | |
| --- | --- | --- | --- | --- | --- | --- | --- | --- | --- | --- |
|  |  |  | mean | S.D. | mean | S.D. | mean | S.D. | mean | S.D. |
| berberine | Day 1 | 8.01 | 5.11 | 2.47 | 0.42 | 0.12 | 148.77 | 72.99 | 121.57 | 17.07 |
|  |  | 24.03 | 8.19 | 3.58 | 0.83 | 0.82 | 358.33 | 119.60 | 554.10 | 251.73 |
|  |  | 72.08 | 13.19 | 5.78 | 0.50 | 0.35 | 218.70 | 118.35 | 306.28 | 94.72 |
|  | Day 28 | 8.01 | 27.12 | 23.31 | 3.67 | 4.48 | 324.10 | 431.97 | 838.44 | 949.05 |
|  |  | 24.03 | 9.51 | 5.23 | 1.33 | 0.47 | 19.10 | 3.28 | 115.09 | 56.44 |
|  |  | 72.08 | 12.92 | 4.60 | 0.50 | 0.35 | 23.83 | 6.25 | 153.27 | 53.73 |
| epiberberine | Day 1 | 0.82 | 4.13 | 0.49 | 0.42 | 0.12 | 9.96 | 4.38 | 11.57 | 1.08 |
|  |  | 2.47 | 4.70 | 1.17 | 0.83 | 0.82 | 30.43 | 15.40 | 72.49 | 26.67 |
|  |  | 7.42 | 10.46 | 5.44 | 2.42 | 2.55 | 17.80 | 7.13 | 128.90 | 48.41 |
|  | Day 28 | 0.82 | 8.39 | 3.84 | 2.17 | 1.43 | 23.34 | 27.62 | 75.82 | 44.51 |
|  |  | 2.47 | 14.71 | 16.38 | 2.08 | 1.53 | 14.50 | 2.54 | 138.58 | 54.58 |
|  |  | 7.42 | 13.06 | 7.00 | 2.67 | 0.94 | 30.37 | 12.39 | 311.25 | 123.81 |
| coptisine | Day 1 | 1.38 | \ | 0.00 | 0.50 | 0.00 | 18.11 | 11.11 | 9.49 | 2.79 |
|  |  | 2.47 | 2.93 | 1.45 | 0.83 | 0.82 | 50.00 | 19.72 | 63.81 | 31.66 |
|  |  | 7.42 | 2.76 | 0.38 | 0.50 | 0.35 | 31.07 | 15.51 | 57.11 | 10.26 |
|  | Day 28 | 1.38 | 8.19 | 6.46 | 0.33 | 0.12 | 52.53 | 72.46 | 99.93 | 135.02 |
|  |  | 2.47 | 10.60 | 4.99 | 1.17 | 0.62 | 2.95 | 2.29 | 4.65 | 4.06 |
|  |  | 7.42 | 4.07 | 2.02 | 0.50 | 0.35 | 2.82 | 0.87 | 10.74 | 6.60 |
| palmatine | Day 1 | 2.03 | 5.96 | 2.81 | 0.42 | 0.12 | 27.93 | 13.97 | 18.08 | 4.07 |
|  |  | 6.09 | 3.29 | 1.62 | 0.83 | 0.82 | 77.60 | 35.30 | 90.31 | 50.76 |
|  |  | 18.26 | 5.18 | 1.21 | 0.50 | 0.35 | 46.60 | 27.16 | 38.00 | 16.92 |
|  | Day 28 | 2.03 | 80.32 | 108.44 | 0.42 | 0.12 | 55.94 | 72.88 | 116.47 | 138.20 |
|  |  | 6.09 | 9.33 | 4.40 | 1.33 | 0.47 | 4.16 | 0.97 | 14.65 | 10.78 |
|  |  | 18.26 | 3.98 | 0.56 | 0.83 | 0.82 | 5.67 | 2.58 | 24.07 | 12.21 |
| jatrorrhizine | Day 1 | 0.31 | 2.64 | 1.25 | 2.33 | 2.59 | 11.04 | 7.79 | 25.63 | 30.02 |
|  |  | 0.93 | 3.57 | 0.37 | 0.83 | 0.82 | 14.64 | 6.91 | 19.94 | 9.16 |

Table. S8 (Continued)

| components | days | Dose  (mg/kg) | T_1/2_  (h) | | T_max_  (h) | | C_max_  (ng/ml) | | AUC_0-t_  (ng/ml*h) | |
| --- | --- | --- | --- | --- | --- | --- | --- | --- | --- | --- |
|  |  |  | mean | S.D. | mean | S.D. | mean | S.D. | mean | S.D. |
|  |  | 2.80 | 3.96 | 0.64 | 0.50 | 0.35 | 14.84 | 10.66 | 22.12 | 4.83 |
|  | Day 28 | 0.31 | 3.39 | 1.60 | 4.17 | 4.17 | 11.15 | 15.03 | 23.94 | 27.68 |
|  |  | 0.93 | 5.33 | 2.53 | 1.00 | 0.71 | 1.29 | 0.31 | 3.87 | 1.24 |
|  |  | 2.80 | 4.98 | 0.56 | 1.33 | 0.47 | 2.08 | 0.41 | 11.36 | 1.79 |
| columbamine | Day 1 | 0.66 | 4.31 | 1.44 | 2.33 | 2.59 | 29.98 | 20.67 | 71.44 | 80.14 |
|  |  | 1.98 | 7.78 | 3.08 | 0.83 | 0.82 | 39.63 | 18.46 | 59.20 | 20.55 |
|  |  | 5.93 | 8.24 | 3.78 | 0.50 | 0.35 | 37.70 | 30.37 | 41.10 | 10.03 |
|  | Day 28 | 0.66 | 10.95 | 7.08 | 0.42 | 0.12 | 30.29 | 40.03 | 68.10 | 76.87 |
|  |  | 1.98 | 10.65 | 3.18 | 1.50 | 1.77 | 1.35 | 0.97 | 9.42 | 7.32 |
|  |  | 5.93 | 12.44 | 3.46 | 1.17 | 0.62 | 2.98 | 0.24 | 12.23 | 4.52 |
